# Untargeted Lipidomics Method for the Discrimination of Five Crab Species by Ultra-High-Performance Liquid Chromatography High-Resolution Mass Spectrometry Combined with Chemometrics

**DOI:** 10.3390/molecules28093653

**Published:** 2023-04-22

**Authors:** Jiaxu Yao, Jinrui Zhu, Minjie Zhao, Li Zhou, Eric Marchioni

**Affiliations:** 1 National Demonstration Center for Experimental Ethnopharmacology Education, School of Pharmaceutical Sciences, South-Central Minzu University, Wuhan 430074, China; 2 Equipe de Chimie Analytique des Molécules Bioactives et Pharmacognoise, Institut Pluridisciplinaire Hubert Curien (UMR 7178, CNRS/UDS), 74 Route du Rhin, 67400 Illkirch, France

**Keywords:** seafood, molecular species, mass spectrum, quantification, identification, lipid

## Abstract

In this study, ultra-high-performance liquid chromatography high-resolution accurate mass-mass spectrometry (UHPLC-HRAM/MS) was applied to characterize the lipid profiles of five crab species. A total of 203 lipid molecular species in muscle tissue and 176 in edible viscera were quantified. The results indicate that *Cancer pagurus* contained high levels of lipids with a docosahexaenoic acid (DHA) and eicosapntemacnioc acid (EPA) structure in the muscle tissue and edible viscera. A partial least squares discriminant analysis (PLS-DA) showed that PE 16:0/22:6, PE P-18:0/20:5, PA 16:0/22:6 and PC 16:0/16:1 could be used as potential biomarkers to discriminate the five kinds of crabs. In addition, some lipids, such as PE 18:0/20:5, PC 16:0/16:1, PE P-18:0/22:6 and SM 12:1;2O/20:0, could be used as characteristic molecules to distinguish between *Cancer magister* and *Cancer pagurus*, which are similar in appearance. This study provides a new perspective on discriminating crab species from MS-based lipidomics.

## 1. Introduction

Crab, as one of the most famous delicacies in the world, is greatly adored by people for its unique taste and distinct flavor [1]. Due to the different consumption habits, crab muscle and edible viscera are individually appreciated. The former is mainly from the claws, legs and abdomen and the latter from the hepatopancreas and gonads [2]. Crab is an excellent source of numerous nutrients essential for human health, including highly unsaturated fatty acids, proteins, minerals and vitamins [3], which bring benefits to humans, such as anti-inflammatory and immunity and cognitive enhancing properties [4].

The 2021 Power of Seafood report published by the Food Industry Association demonstrated that the overall seafood category was up 28.4% in retail around the world. In China, the demand for crab is also constantly increasing, as evidenced by the growth of crab farming from 821,000 tons in 2010 to 1,063,000 tons in 2020. Crab ranks third in global seafood production after shrimp and lobsters and has significant commercial value [5,6]. Mainly, crab is one of the most diverse of the decapod crustaceans, which can be found in both fresh and ocean waters [7]. The nutritional value of different crabs might be different due to the fact of their different nutritional components.

It has been documented that approximately 7000 species of crab survive throughout the world, either in freshwater lakes or in the ocean [8]. However, the majority of crabs are used for food, whether they are raised or wild. Commercially valuable crabs can be easily purchased for consumption, such as red king crab (*Paralithodes camtschaticus*), swimming crab (*Portunus trituberculatus*), Chinese mitten crab (*Eriocheir sinensis*), Dungeness crab (*Cancer magister*) and brown edible crab (*Cancer pagurus*) [9].

Lipids, a major constituent of various foods, play vital roles in many cellular processes, such as energy storage, signal-mediated processes and tumor suppression [10,11,12]. Lipids contribute to the quality features of food products, including flavor and nutritional value. Numerous studies have focused on lipids, resulting in the emergence of lipidomics. Lipidomics, a branch of metabolomics, has superiority in providing lipid profiles of biological samples, especially those rich in lipids. In nutritional research, lipidomics has been routinely employed to elucidate the interactions among diet, nutrients and human metabolism to optimize food processing and to evaluate the nutrition of foods [13]. Consumers can establish guidelines for personalized nutrition based on lipid information [14].

Statistically, the number of publications on the lipidomics of marine animals showed an explosive increase over the past few years [15], and numerous studies have been conducted concerning the lipids of crabs. The research objectives are also focused on improving the level of beneficial lipids or survival rates by changing the dietary structure of crabs [16,17] and analyzing the lipid profile of crabs to determine their nutritional information [18]. Nevertheless, few studies have been performed comparing the lipids of multiple crab species. Thus, it is meaningful to comprehensively characterize and compare the lipid compositions among crab species from a lipidomics perspective.

In recent years, one of the most valuable technologies for lipid identification has been ultra-high-performance liquid chromatography coupled to high-resolution mass spectrometry [19], which possesses the advantages of accurate quality and excellent sensitivity. Quadrupole-Exactive high-resolution accurate mass spectrometry (HRAM) is a developed technique that can provide complete lipid molecular information without derivatization [20]. Thus, this study aimed to identify the lipid classes and lipid molecular species of the muscles and edible viscera tissue from five kinds of edible crabs by a UHPLC-HRAM/MS approach. Subsequently, the different compositions of the lipid profiles were analyzed by lipidomics combined with chemometrics. This is the first investigation involving an in-depth and comprehensive identification and comparison of the lipid profiles of different crabs. This study may help us better understand the nutritional values of edible crabs.

## 2. Results and Discussion

### 2.1. Total Lipid Content of Crab Muscle and Edible Viscera

The total lipid content of crab muscle and edible viscera are shown in Table S2. No significant difference (*p* > 0.05) was exhibited in the content of the crab muscle lipids. As for edible viscera, the five crabs exhibited a significant difference (*p* < 0.05). The total lipid content of the edible viscera from *P. camtschaticus* (766.3 mg/g) and *E. sinensis* (761.0 mg/g) showed a higher level than the other three crab lipids. Overall, the total lipid content of the edible viscera was higher than that in the muscles, which is consistent with the results of previous reports [2,21]. The reason for this is probably that crab muscles mainly store protein, while the viscera stores fat and cholesterol. The high level of total lipids in the crab samples results in biological weight gain, which is associated with consuming crab-containing diets [22]. In addition, many factors might affect the lipid content of different crab species, such as temperature [23], maturity [24] and diets [25]. The variation of the lipid content in crabs under different physiological conditions requires further experimental analysis.

### 2.2. Validation of the UHPLC-HRAM/MS Method

As described in this method, all lipids were quantified by the internal standard method. The internal standards were mixed with each test sample to quantify the lipid molecules, so the relative standard deviation (RSD) values of the peak areas of the internal standards could be used as a measure of the stability of the UHPLC-HRAM/MS instrument during the measurement of the sample [26]. The RSD values of the peak area of the internal standard are displayed in Figure S1. It was found that the RSD values of 84.3% of features were less than 20%, which demonstrated that the signal was stable during the sample detection.

Further validation was carried out using seven internal standards. The linear regression equations derived from the different concentrations of the seven lipid standards are shown in Table S3, which reveals an excellent linearity (R^2^  ≥  0.990). The limit of detection (LOD) value was less than 2.28 ng/mL, and the limit of quantification (LOQ) values were between 0.99 ng/mL and 7.52 ng/mL.

### 2.3. Lipid Identification

**Figure 1:**
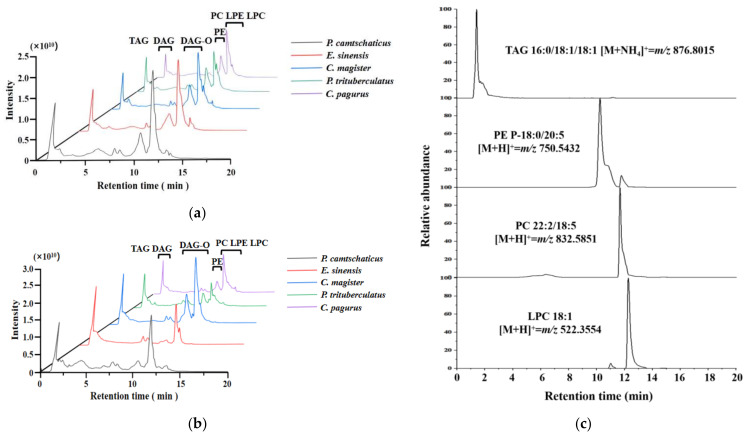
Total ion chromatograms of lipids in the positive ion mode from the (**a**) muscle and (**b**) edible viscera of five crabs. (**c**) Extracted individual ion chromatograms of the muscle lipids of *C. magister*, showing the species TAGs of 16:0/18:1/18:1, PE P-18:0/20:5, PC 22:2/18:5 and LPC 18:1.

The muscle lipids and edible viscera lipids of the five crabs were identified by UHPLC-HRAM/MS, with the electrospray ionization (ESI) source in positive and negative ion modes. Different lipid classes were detected in specific ESI source patterns due to the fact of their polarities and electric charges [27]. The PC, LPC, PE, LPE, TAG and DAG were analyzed under the positive ion mode ([M + H]^+^ and [M + NH_4_]^+^), while the remaining lipids were examined under the negative ion mode ([M − H]^−^, [M + HCOO^−^]^−^ and [M − 2H]^2−^). The lipid profiles of the muscle (Figure 1a) and edible viscera (Figure 1b) from five crab species could be detected by ESI sources in the positive ion mode within 20 min. From the total ion chromatograms plot in the positive ion mode, the weakly polar lipid such as TAG were retained for a short time, and the retention times of the molecular species in the same lipid classes were similar. However, the different peak shapes indicate that that the contents of common lipid classes could be different in these crab samples. Figure 1c presents an example of extracted ion chromatograms (EICs) of *m/z* 876.8015, 750.5432, 832.5851 and 522.3554 from *C. magister* muscle lipids.

The lipid molecular species were identified by MS/MS spectrometry on the basis of their characteristic mass values and fragment ions. Lipid classes can be classified by the types of polar headgroups, which exhibit characteristic fragments in the MS/MS spectrum. For example, *m/z* 184.0730 [C_5_H_15_NO_4_P]^+^ is the typical fragment of the choline headgroup of PC, and *m/z* 241.0130 [C_6_H_10_O_8_P]^−^ is the polar head of PI. In addition, other MS/MS fragments can provide information to infer the fatty acyl group of the lipids and the total relative molecular masses.

**Figure 2:**
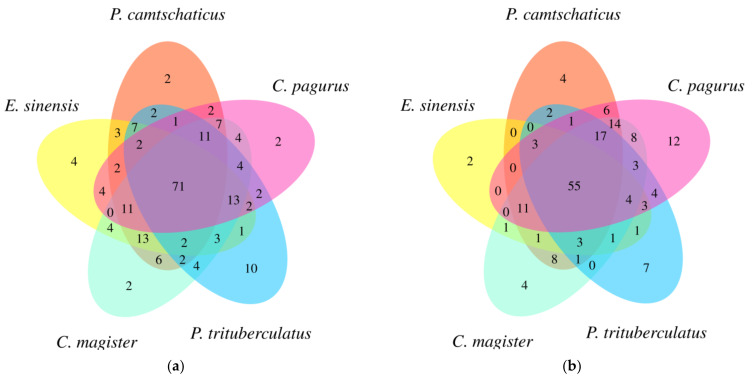
Distribution of lipid molecular species in the (**a**) muscle and (**b**) edible viscera of five crabs.

A total of 14 lipid subclasses and 203 molecular species were determined in the muscle lipids (Table S4) and 13 lipid subclasses and 176 molecular species in the edible viscera lipids (Table S5). Seventy-one and fifty-five common molecular species were detected, respectively, in the muscles and edible viscera of the five crabs (Figure 2a,b). The concentrations of each lipid subclass in the crab muscles and edible viscera are displayed in Tables S6 and S7, respectively. The proportion of each lipid subclass is calculated and shown in Figure S2. PE, PC and TAG were the main lipid subclasses in the crab muscle samples, while TAG was the predominant one in the edible viscera of *E. sinensis*, which accounted for more than 89%. These results are similar to that published by Wang et al. [28], who illustrated that crabs stored great numbers of TAGs in visceral organs for energy expenditure during starvation, molting or reproduction [29,30]. Especially, a relatively high level of PA was found in the crab muscles, which was not encountered in previous studies. According to research, more than 70% of PA is consumed at the time crabs mature from larvae [31]. The absence of PA might be one of the causes of the high mortality of crab larvae, so diets adding relevant lipids would be used to increase the survival rate of crabs. In comparison, phospholipids have a crucial effect on cell membranes, mainly by maintaining endogenous systems, serving as binding sites for proteins and participating in signaling [32], which play a vital role in the growth and metabolism of crabs [33,34]. Moreover, they are precursors of essential steroids [35].

It is well known that the lipids containing long-chain polyunsaturated fatty acyl chains (LC-PUFAs), such as EPA and DHA, play an irreplaceable role in brain development [36] and have properties such as antitumor [37] and anti-inflammatory [5]. In addition, LC-PUFAs exhibit better bio-efficacy and bioavailability than other lipids [38]. In this study, some DHA/EPA-TAG and DHA/EPA-PL molecules were detected in the muscle and edible viscera of the five crab species. As shown in Figure S3, the contents of DHA/EPA-PLs and DHA/EPA-TAGs (DHA/EPA-PLs + DHA/EPA-TAGs) were the highest (72.26%) in *C. pagurus* muscle, while they appeared to have the lowest levels in *E. sinensis* edible visceral tissue. The total contents of DHA/EPA-PLs and DHA/EPA-TAGs were the highest in *C. pagurus*, with significant differences (*p* < 0.05) compared to the other groups. The DHA/EPA distribution in PLs and TAGs was consistent with the overall fatty acid composition of the plentiful PUFAs in marine animals and plant plankton [39]. Herein, our results indicate that crab muscles and edible viscera are an excellent source of DHA/EPA.

**Figure 3:**
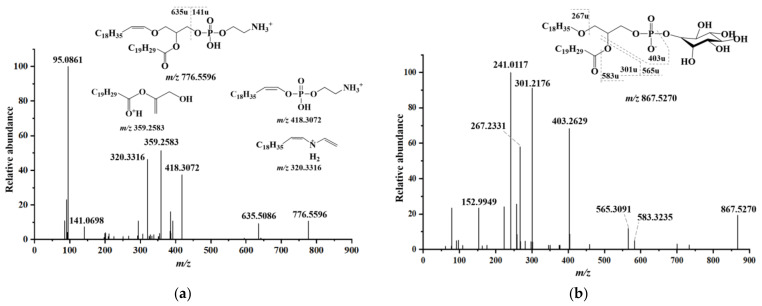

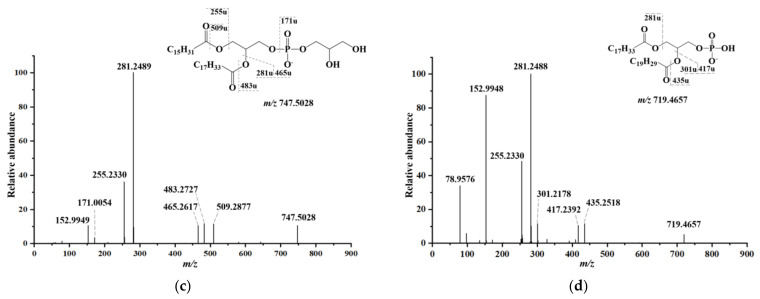
MS/MS fragmentation pathway of (**a**) PE P-20:1/20:5 (*m/z* 776.5596) under a positive ion mode and (**b**) PI O-16:1/20:5 (*m/z* 867.5270), (**c**) PG 16:0/18:1 (*m/z* 747.5028) and (**d**) PA 18:1/20:5 (*m/z* 719.4657) under a negative ion mode.

Furthermore, ether-linked phospholipids were found in the lipids of the muscle and edible viscera of the crabs, which are essential signaling molecules needed for the execution of complex life activity [40,41]. A representative MS/MS fragmentation spectrum in the positive ion mode of PE P-20:1/20:5 (*m/z* 776.5596) from *P. camtschaticus* muscle is shown in Figure 3a. This molecular species only existed in the *P. camtschaticus* muscle and was most abundant (3183 nmol/g) in plasmalogen PE. The product ion at *m/z* 141.0698 (C_2_H_8_NO_4_P) was the characteristic fragment of the PE molecule. The product ion at *m/z* 635.5086 corresponded to the loss of the ethanolamine headgroup. The oxygen at the sn-1 position attacked the phosphorus atom, which led to the formation of a new oxygen–phosphorous bond. At the same time, hydrogen was extracted from C-2 of the glycerol backbone, forming a double bond between C-1 and C-2 of the glycerol backbone [42,43]. Therefore, the ion fragment at *m/z* 418.3072 had the structure of 20:1 ether and C_2_H_8_NO_3_P, and the ion fragment at *m/z* 320.3316 was a neutral loss corresponding to H_3_PO_4_ on this basis.

The representative MS/MS fragmentation spectrum in the negative ion mode of PI O-16:1/20:5 (*m/z* 841.5156) from *C. magister* edible viscera is provided in Figure 3b. The product ion at *m/z* 301.2182 corresponds to the ion of EPA, and *m/z* 539.2991 corresponds to the loss of EPA [44]. The ion fragment at *m/z* 377.2467 was identified as the residue fragment, which was generated by the loss of the inositol headgroup of the ion fragment at *m/z* 539.2991. The ion fragment at *m/z* 557.3096 corresponded to the loss of C20:5 acyl group from the precursor ion. The ion fragment at *m/z* 241.0117 and 152.9948 were characteristic signals of the PI molecules, which were generated from the inositol headgroup. The former was a structure formed by dehydrating the head bond, while the latter was a five-membered ring structure formed by disconnecting two fatty acid chains and a six-membered ring.

PG 16:0/18:1 was detected in the muscles of all five kinds of crab, and the fragmentation pathways of PG 16:0/18:1 are displayed in Figure 3c. The ion fragment at *m/z* 747.5028 corresponds to the empirical formula C_40_H_76_O_10_P. The ion fragment at *m/z* 171.0054 was the C_3_H_7_O_6_P structure, which was the head group of the PG molecule, and the ion fragment at *m/z* 152.9949 was a five-membered ring structure formed by dehydration on this basis. As seen in Figure 3d, the PA 18:1/20:5 from *P. camtschaticus*, which had the highest level (750.6 ± 41.4 nmol/g) among all species, displayed signals at *m/z* 719.4567 in the MS^−^ spectrum, which corresponded to the empirical formula C_41_H_69_O_8_P^−^. The ion fragments at *m/z* 281.2490 and *m/z* 301.2176 were oleic acid and EPA, respectively. The ion fragments at *m/z* 417.2411 and 4435.2516 were derived from the loss of the EPA and C20:5 acyl groups, respectively, from the precursor molecule.

### 2.4. Multivariate Analysis

**Figure 4:**
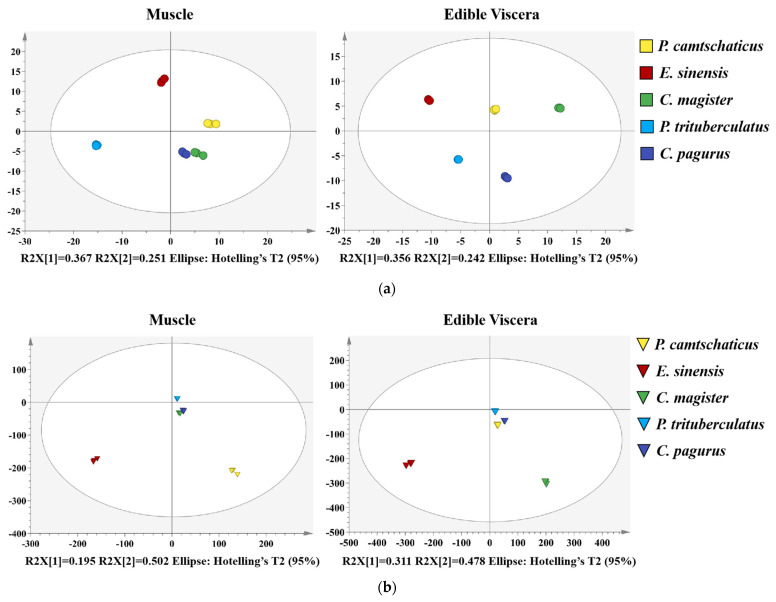

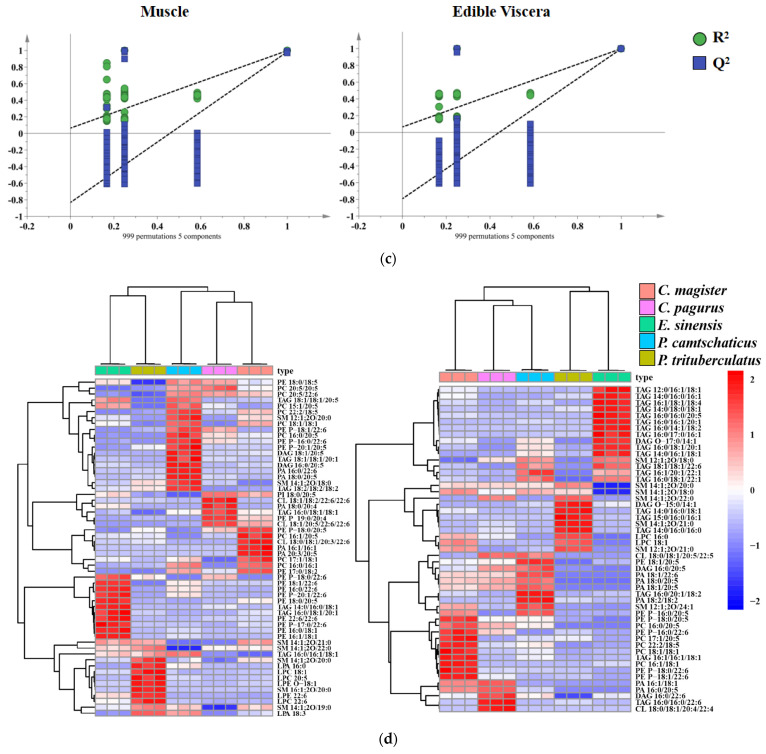
(**a**) PCA score plot of identified lipids in muscle with R^2^X = 0.994 and Q^2^X = 0.982 and edible viscera with R^2^X = 0.997 and Q^2^X = 0.985. (**b**) PLS-DA score plot of identified lipids in muscle with R^2^X = 0.998, R^2^Y = 0.999 and Q^2^ = 0.997 and edible viscera with R^2^X = 0.998, R^2^Y = 0.999 and Q^2^ = 0.998. (**c**) Permutation constructed on the basis of the PLS-DA of the contribution molecular species in muscle and edible viscera, and (**d**) Hierarchical cluster dendritic diagram of muscle and edible viscera and hierarchical cluster analysis based on molecular species in muscle and edible viscera with VIP > 1. Colors represent different concentrations indicated by the color bar.

As MS-based lipidomics generates a large amount of data in the analysis process [45], principal component analysis (PCA), a multivariate statistical method, was used to examine the correlations among the lipidomics data with multiple variables and to compare the lipid molecules and contents of the different crabs to facilitate the analysis and visualization. Figure 4a presents the PCA score plots (R^2^X = 0.994, Q^2^X = 0.982) of the crab lipids in the different muscles, displaying the clustering of each sample in the first two principal component (t1 and t2) score plots, accounting for 0.367 and 0.251 of the total variance, respectively. The PCA score plots (R^2^X = 0.997, Q^2^X = 0.985) of the edible visceral lipids in the different crabs indicate that the first two principal components (t1 and t2) accounted for 0.356 and 0.242 of the total variance. From the PCA score plot (Figure 4a), the five crab types were nonoverlapping with each other, which indicates that the metabolites of each crab were discrepant. Therefore, the PCA model had good separation and clustering results and can be used as a method to effectively distinguish different samples.

Furthermore, the separations were tested in the PLS-DA score plot. R^2^ represents the degree of fitting between the model and crab lipid data, and Q^2^ (cumulative) represents the prediction ability of the model for new data [46]. For the crab muscle samples, the PLS-DA score plot is displayed in Figure 4b (R^2^X = 0.998, R^2^Y = 0.999, Q^2^ = 0.997), which demonstrates a good separation effect and prediction ability. After 999 permutations (Figure 4c), the values of R^2^ = (0.0, 0.062) and Q^2^ = (0.0, −0.823) of this model were more prominent than the random values of all samples, which is similar to the PLS-DA score plot of the edible visceral samples. All these data underlie the classification of the five crab species and show a good predictive ability for a new data set.

Subsequently, the value of VIP (variable importance in projection) was applied to screen for significant differences in the lipids of the five crab species, which was available from the PLS-DA model. In the crab muscle model, a total of 57 lipid molecules with VIP > 1 were analyzed by cluster analysis to assess the similarity of the crab samples (Figure 4d). According to the dendritic diagram, *C. magister* and *C. pagurus* was in one group. At the same time, 51 lipid molecules met VIP > 1, and *C. pagurus* and *P. camtschaticus* belonged to one group based on the similarities in the composition of the edible viscera. The results were also well reflected in the PCA diagram. Thus, studying the pedigree relationship of lipidomics characteristics can provide a reference for species classification.

Additionally, one-way analysis of variance (ANOVA) was conducted to determine the final statistically significant (*p* < 0.05) lipid species. In general, markers need to meet *p* < 0.05 and VIP > 1 [47], which could be identified as potentially characteristic metabolites, thus achieving the ability to identify species accurately. In the crab muscle model, 26 lipid molecules were selected as markers (Table S8), such as PE 16:0/22:6, PE P-18:0/20:5, PA 16:0/22:6 and PC 16:0/16:1. The distribution of 26 lipids in the muscle content of the five species of crab is shown in Figure S4a. For example, when the detected range of PE 16:0/22:6 was 8000 nmol/g, the crab species can be judged as *E. sinensis*. In the samples of edible viscera, 17 lipid molecules were selected as markers (Table S8), including TAG 16:0/20:1/18:2, SM 14:1;2O/22:0, PE 18:1/20:5 and TAG 16:0/18:1/20:1, whose distributions are shown in Figure S4b.

### 2.5. Orthogonal Partial Least Squares Discrimination Analysis

Due to the highly similar appearance of *C. magister* and *C. pagurus*, the two species were often confused without relevant books and professionals. Here, the differences between the two crabs were analyzed from a lipidomics perspective combined with a metrological approach.

**Figure 5:**
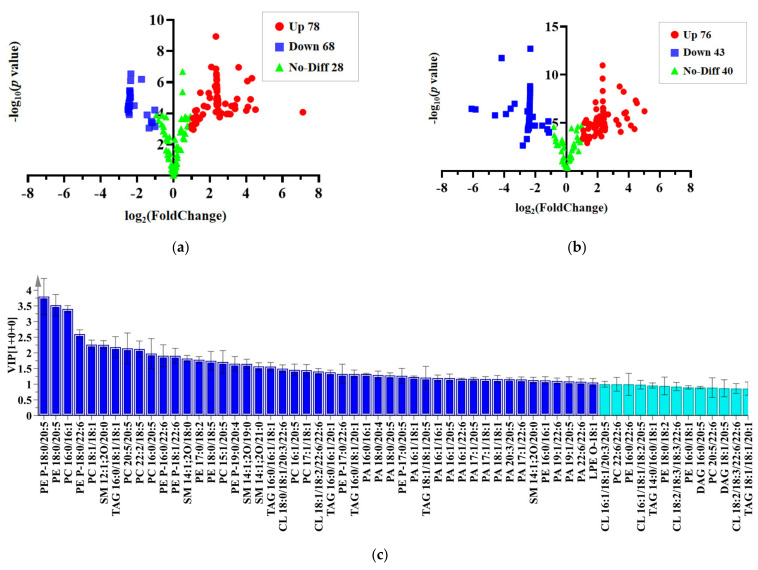

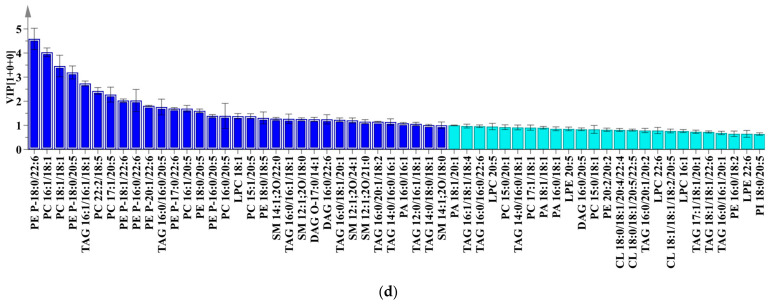
An orthogonal partial least squares discrimination analysis was performed for *C. magister* and *C. pagurus*. Volcano plots of lipids from (**a**) muscle and (**b**) edible viscera. VIP maps of lipid molecules in (**c**) crab muscle and (**d**) edible viscera. Dark blue indicates VIP > 1.

In this study, the principle for the selection of significantly different lipid species between *C. magister* and *C. pagurus* is a sufficiently high variable importance for the projection (VIP > 1.3) and the univariate statistical analysis, including a fold change (log_2_(FC) ≥ 1 or ≤−1) and T-test criteria (*p* < 0.05). Overall, 174 lipids from muscles were calculated and are shown in volcano plots in Figure 5a, among which 78 lipids were upregulated and 68 were downregulated in *C. magister* compared with *C. pagurus*. Analogously, Figure 5b demonstrates that 76 lipids from edible viscera were upregulated and 43 were downregulated. In addition, the VIP values of each lipid molecule were obtained from orthogonal partial least squares discrimination analysis (OPLS-DA). Figure 5c,d represent the VIP value map of some quantified metabolites from the muscles and edible viscera, respectively. Finally, 20 lipid molecules from muscles and 17 lipid molecules from edible viscera were selected as the characteristic molecules to distinguish *C. magister* and *C. pagurus* (Table 1).

## 3. Materials and Methods

### 3.1. Ethical Statement

In this study, all experimental animals were adequately cared for, their pain minimized, and killed painlessly. In addition, the number of all animals was controlled to the minimum required to obtain scientific results.

### 3.2. Sample Preparation

Five living female farmed crab species (Figure S5), including *P. camtschaticus* (*Paralithodes camtschaticus*), *E. sinensis* (*Eriocheir sinensis*), *C. magister* (*Cancer magister*)*, P. trituberculatus* (*Portunus trituberculatus*) and *C. pagurus* (*Cancer pagurus*), were purchased from a local seafood market in Wuhan, China, in October 2020, with the help of professionals (12 fresh crabs of each species, individually weighed, *P. camtschaticus*: 1500–1700 g; *E. sinensis*: 150–200 g; *C. magister*: 750–800 g; *P. trituberculatus*: 250–300 g; and *C. pagurus*: 900–1000 g). The ages of each species of crab shell were similar, and all crab limbs were free of damage. According to a previous study [48], all crabs were electrocuted through 110 volts and 2 amps of current for 10 s with a Crustastun machine (Studham Technologies, Scotland, UK) and killed immediately. The edible viscera and muscle were manually separated from the crabs, immediately preserved in liquid nitrogen and then lyophilized using a vacuum freeze-dryer (Labconco Corporation, Kansas, MO, USA) with a vacuum of 0.06 MPa. The freeze-dried crab tissues were stored at −80 °C until analysis.

### 3.3. Standards and Reagents

The PL standards were purchased from Avanti Polar Lipids, including phosphatidylcholine (PC 17:0/17:0), phosphatidylethanolamine (PE 17:0/17:0), phosphatidylglycerol (PG 17:0/17:0), phosphatidic acid (PA 17:0/17:0), phosphatidylinositol (PI 8:0/8:0), sphingomyelin (SM d18:1/12:0) and SPLASH LIPIDOMIX (isotopic internal standard mixture). Glyceryl trilinoleate (TAG) was purchased from Sigma Aldrich. Other chemicals, including methanol, chloroform, acetonitrile and ammonium formate, were purchased from Macklin Biochemical Technology Co., Ltd. (Shanghai, China) (high-performance liquid chromatography or analytical reagent grade).

### 3.4. Lipid Extraction

All biological replicates were sourced from 12 crabs of the counterpart species, and a triplicate analysis was performed for each crab species. Total lipids were extracted using the methods described by Yang et al. with minor modifications [49]. Briefly, the freeze-dried edible viscera and muscle tissues were weighed and homogenized in 10 mL of methanol in a homogenizer (Bead Mill 4, Fisherbrand, Tokyo, Japan) for 30 s (×2 cycles). Subsequently, 10 mL of the homogenate (1 g) was transferred to a beaker, and 20 mL of chloroform was added. The mixture was placed in an ultrasonic bath and processed for 30 min at 20 °C. Then, the mixture was centrifuged at 4000× *g* for 10 min. The supernatant was transferred to a clean bottle, and the lower phase was re-extracted twice with chloroform:methanol (2:1, *v/v*). Finally, the organic solvents were combined and evaporated under a vacuum rotary evaporator (40 °C, 350 kPa). The residue was further dried using a gentle stream of nitrogen. Dried lipid extracts were weighted and stored at −20 °C for further analysis by UHPLC-HRAM/MS. All analyses were completed within two weeks after extraction.

### 3.5. Lipid Identification and Semi-Quantification

Lipid molecular species were identified with MS-DIAL software (version 4.48) and Xcalibur 4.0 software (ThermoFisher, Waltham, MA, USA), as well as the LipidMaps and LipidBank websites. False positive data were manually excluded based on database matching and fragmentation information.

Seven PL standards, including PC 17:0/17:0 (standard for PC and lysophophatidylcholine (LPC)), PE 17:0/17:0 (standard for PE and lysophosphatidylethanolamine (LPE)), PG 17:0/17:0 (standard for PG, lysophosphatidylglycerol (LPG) and cardiolipin (CL)), PA 17:0/17:0 (standard for PA and lysophosphatidic acid (LPA)), SM d18:1/12:0 (standard for SM), PI 8:0/8:0 (standard for PI and lysophosphatidylinositol (LPI)) and TAG 18:2/18:2/18:2 (standard for TAG and diacylglycerol (DAG)), were used for the construction of the calibration curves. These standards were quantitatively dissolved in a chloroform:methanol (2:1, *v/v*) mixture as stock solutions and then diluted into a series of concentrations, with a constant concentration of internal standards from SPLASH LIPIDOMIX (as displayed in Table S1 of the Supplementary Materials), including PC 15:0/18:1(d7), PG 15:0/18:1(d7), PE 15:0/18:1(d7), TAG 15:0/18:1(d7)/15:0, PI 15:0/18:1(d7), SM d18:1/18:1(d9) and PA 15:0/18:1(d7). The internal standard method was applied to quantify the lipid concentrations of crab samples [50]. Briefly, the same concentration of internal standards was added to each crab lipid, and the samples were injected for detection. The crab lipids were quantified according to the calibration curves of the peak area ratio of the standard and internal standard within the same class so the concentration (mg/mL) of lipids could be obtained. Subsequently, the concentration (nmol/g) of each lipid molecule in a dried biological sample was available by calculation. Furthermore, all molecular quantifications were based on the EICs of individual lipids, with *m/z* expansion at ±5 ppm, and all crab samples were analyzed with three duplicate samples.

### 3.6. UHPLC-HRAM/MS Analysis

#### 3.6.1. UHPLC Conditions

The total lipids of the different crabs were identified using a UHPLC system (Dionex, UltiMate, 3000 RSLC) coupled with QE Orbitrap/MS (ThermoFisher Scientific, Bremen, Germany). A BEH-HILIC column (100 × 1.0 mm, 1.7 μm, Sigma–Aldrich/Supelco, Bellefonte, PA, USA) was used for chromatographic separation. The flow rate was 0.1 mL /min, and the column temperature was 40 °C. Eluent A and eluent B were 5 mM ammonium formate in water and in acetonitrile, respectively. The chromatographic gradient elution mode was as follows: 0 min, 5% B; 4 min, 5% B; 10 min, 40% B; 15 min, 40% B; 16 min, 5% B; and 20 min, 5% B. The injection volume was 2 μL.

#### 3.6.2. Quadrupole-Exactive High-Resolution Accurate Mass Spectrometry

The desolvation ESI source parameters were set as follows—electrospray voltage: 3.2 kV; a sheath gas flow rate: 35 arbitrary units (arb unit); auxiliary gas flow rate: 10 arbs; capillary temperature: 325 °C; heater temperature: 350 °C; collision energy: 35 eV; dynamic exclusion: 10 s; and isolation window: 3.0 *m/z*. The data were acquired in the positive ionization mode (*m/z* 120–1800) and negative ionization mode (*m/z* 120–1800) with dependent MS/MS acquisition. The resolutions of the full-scan spectra and the fragment spectra were 140,000 and 70,000, respectively. All lipid identifications were determined by MS and MS/MS, with an MS mass error of <5 ppm and MS/MS mass error of <8 ppm.

To investigate the background contamination and the method’s validation, quality control procedures were applied to this experiment, which contained solvent blanks, solvent spiked with internal standards and matrix blanks without spiking the internal standards [51]. To test the stability, the RSD values of the peak areas of the internal standards in three parallel samples were calculated. The LOD and LOQ were the concentrations of the standards corresponding to the signal-to-noise (S/N) values of 3 and 10 [52].

### 3.7. Statistical Analysis

The data are expressed as the mean ± SD. SPSS software (version 24.0) was used for the one-way analysis of variance, and *p* < 0.05 was considered statistically significant. Lipid molecule species were identified and quantified with MS-DIAL software (version 4.48) and Xcalibur 4.0 software (ThermoFisher, Waltham, MA, USA), as well as the LipidMaps and LipidBank websites. MetaboAnalyst 5.0 was used for the cluster analysis, and SIMCA 14.1 was used for the PCA, PLS-DA and OPLS-DA.

## 4. Conclusions

This study applied the UHPLC-HRAM/MS approach to discriminate the lipid molecules in the muscles and edible viscera of five kinds of crabs, including *P. camtschaticus*, *E. sinensis*, *C. magister*, *P. trituberculatus* and *C. pagurus*. Combined with chemometric analysis, 14 lipid subclasses in muscle tissue and 13 lipid subclasses in edible viscera were detected and quantified. *C. pagurus* had the highest DHA/EPA-PL molecule content in the muscle and edible viscera tissue of five crab species. Our work can help researchers better understand the lipid composition of crab muscle and edible visceral tissues and better select or assess their nutritional values.

In addition, we developed a new way to distinguish two kinds of crabs with similar appearances (*C. magister* and *C. pagurus*) based on lipidomics. In the muscle samples, twenty characteristic lipids were screened, such as PE 18:0/20:5, PC 16:0/16:1, PE P-18:0/22:6 and SM 12:1; 2O/20:0, as the basis for distinguishing *C. magister* and *C. pagurus*. Seventeen characteristic lipids, such as PE P-18:0/22:6, PC 16:1/18:1, PC 18:1/18:1 and PE P-18:0/20:5, were screened from the edible visceral samples. However, our work focused on the differences in the crab varieties, while some factors that might affect the lipid composition, such as maturity and living environment, were not considered. Therefore, the effects of various factors on the lipid composition of crabs will be investigated in further study.

## Figures and Tables

**Table 1:** The significantly different lipids in the muscles and edible viscera of *C. magister* and *C. pagurus*.

Muscle				Edible Viscera			
Compound	VIP	log_2_(FC)	*p*-Value	Compounds	VIP	log_2_(FC)	*p*-Value
PE 18:0/20:5	3.28	2.41	<0.01	PE P-18:0/22:6	4.43	2.49	<0.01
PC 16:0/16:1	3.28	4.06	<0.01	PC 16:1/18:1	3.97	2.39	<0.01
PE P-18:0/22:6	2.37	−1.75	<0.01	PC 18:1/18:1	3.37	3.49	<0.01
SM 12:1;2O/20:0	2.21	2.39	<0.01	PE P-18:0/20:5	3.16	1.20	<0.01
PC 18:1/18:1	2.08	1.94	<0.01	TAG 16:1/16:1/18:1	2.68	5.02	<0.01
PC 20:5/20:5	1.96	−1.16	<0.01	PC 22:2/18:5	2.33	2.14	<0.01
PC 22:2/18:5	1.93	1.20	<0.01	PC 17:1/20:5	2.23	4.38	<0.01
PE P-18:1/22:6	1.78	−2.46	<0.01	PE P-16:0/22:6	2.04	1.14	<0.01
SM 14:1;2O/18:0	1.78	−2.40	<0.01	PE P-18:1/22:6	1.95	1.88	<0.01
PE 17:0/18:2	1.61	1.48	<0.01	PE P-20:1/22:6	1.76	3.68	<0.01
PC 15:1/20:5	1.56	−1.22	<0.01	TAG 16:0/16:0/20:5	1.70	1.81	<0.01
SM 14:1;2O/19:0	1.56	2.85	<0.01	PE P-17:0/22:6	1.65	3.42	<0.01
PE P-19:0/20:4	1.52	−1.03	<0.01	PC 16:1/20:5	1.65	3.36	<0.01
SM 14:1;2O/21:0	1.51	4.24	<0.01	PE 18:0/20:5	1.54	1.92	<0.01
CL 18:0/18:1/20:3/22:6	1.46	2.44	<0.01	PE P-16:0/20:5	1.36	3.73	<0.01
TAG 16:0/16:1/18:1	1.45	−2.36	<0.01	LPC 18:1	1.33	1.91	<0.01
PC 17:1/18:1	1.41	7.12	<0.01	PC 15:1/20:5	1.33	2.21	<0.01
CL 18:1/18:2/22:6/22:6	1.37	−2.39	<0.01				
PC 16:1/20:5	1.36	2.33	<0.01				
TAG 16:0/16:1/20:1	1.35	2.40	<0.01				

VIP: variable importance in projection; FC: fold change.

## Data Availability

The data that support the findings of this study are available on request from the corresponding author.

## Supplementary Materials

The following supporting information can be downloaded at: https://www.mdpi.com/article/10.3390/molecules28093653/s1, Figure S1: RSD distribution of the peak area of seven internal standards in 10 groups of different samples; Figure S2: Lipid composition (%) in the muscles and edible viscera of five crab species; Figure S3: The percentage contents of DHA/EPA-PLs and DHA/EPA-TAGs in the total lipids extracted from crab muscle and edible viscera. The data are expressed as the mean ± SD. Different letters indicate a significant difference (*p* < 0.05) among the different samples; Figure S4: Boxplots of the significantly different lipids in the muscles (A) and edible viscera (B) of five crab species; Figure S5: Optical images of the crab species; Table S1: Concentration (ng/mL) of 7 lipid standards and corresponding internal standard (IS) for the construction of calibration curves; Table S2: Total lipids content in five kinds of crabs; Table S3: The linear regression equations, linear regression coefficients (R^2^), LOD and LOQ of lipid standards; Table S4: Molecular species of lipids extracted from the muscles of five kinds of crabs; Table S5: Molecular species of lipids extracted from the edible viscera of five kinds of crabs; Table S6: Concentrations of the different lipid subclasses in the five kinds of crab muscles (nmol/g); Table S7: The concentrations of different lipid subclasses of the five kinds of edible viscera (nmol/g); Table S8: The significantly different lipids in the muscle and edible viscera of five crab species.

## References

[1] Yang F., Guo H., Gao P., Yu D., Xu Y., Jiang Q., Yu P., Xia W. (2021). Comparison of methodological proposal in sensory evaluation for Chinese mitten crab (*Eriocheir sinensis*) by data mining and sensory panel. Food Chem..

[2] Lu T., Shen Y., Cui G., Yin F., Yu Z., Zhou D. (2020). Detailed Analysis of Lipids in Edible Viscera and Muscles of Cooked Crabs *Portunus trituberculatus* and *Portunus pelagicus*. J. Aquat. Food Prod. Technol..

[3] Barrento S., Marques A., Teixeira B., Anacleto P., Vaz-Pires P., Nunes M.L. (2009). Effect of season on the chemical composition and nutritional quality of the edible crab Cancer pagurus. J. Agric. Food Chem..

[4] Nanda P.K., Das A.K., Dandapat P., Dhar P., Bandyopadhyay S., Dib A.L., Lorenzo J.M., Gagaoua M. (2021). Nutritional aspects, flavour profile and health benefits of crab meat based novel food products and valorisation of processing waste to wealth: A review. Trends Food Sci. Technol..

[5] Narayanasamy A., Balde A., Raghavender P., Shashanth D., Abraham J., Joshi I., Nazeer R.A. (2020). Isolation of marine crab (*Charybdis natator*) leg muscle peptide and its anti-inflammatory effects on macrophage cells. Biocatal. Agric. Biotechnol..

[6] Venugopal V., Gopakumar K. (2017). Shellfish: Nutritive Value, Health Benefits, and Consumer Safety. Compr. Rev. Food Sci. Food Saf..

[7] Yeo D.C.J., Ng P.K.L., Cumberlidge N., Magalhães C., Daniels S.R., Campos M.R. (2007). Global diversity of crabs (Crustacea: Decapoda: Brachyura) in freshwater. Hydrobiologia.

[8] Ng P.K.L. (2017). Collecting and processing freshwater shrimps and crabs. J. Crust. Biol..

[9] Ferdoushi Z., Zhang X.G., Hasan M.R. (2010). Mud crab (*Scylla* sp.) marketing system in Bangladesh. Asian J. Food Agro-Ind..

[10] Yao Y., Ding L., Huang X. (2020). Diverse Functions of Lipids and Lipid Metabolism in Development. Small Methods.

[11] Welte M.A., Gould A.P. (2017). Lipid droplet functions beyond energy storage. Biochim. Biophys. Acta Mol. Cell. Biol. Lipids.

[12] Casas-Agustench P., Cherubini A., Andres-Lacueva C. (2017). Lipids and physical function in older adults. Curr. Opin. Clin. Nutr. Metab. Care.

[13] Hyötyläinen T., Bondia-Pons I., Orešič M. (2013). Lipidomics in nutrition and food research. Mol. Nutr. Food Res..

[14] Castro-Alves V., Orešič M., Hyötyläinen T. (2022). Lipidomics in nutrition research. Curr. Opin. Clin. Nutr. Metab. Care.

[15] Imbs A.B., Ermolenko E.V., Grigorchuk V.P., Sikorskaya T.V., Velansky P.V. (2021). Current Progress in Lipidomics of Marine Invertebrates. Mar. Drugs.

[16] Yuan Y., Xu F., Jin M., Wang X., Hu X., Zhao M., Cheng X., Luo J., Jiao L., Betancor M. (2021). Untargeted lipidomics reveals metabolic responses to different dietary n-3 PUFA in juvenile swimming crab (*Portunus trituberculatus*). Food Chem..

[17] Ding Z. (2021). Lipid metabolism disorders contribute to the pathogenesis of *Hepatospora eriocheir* in the crab *Eriocheir sinensis*. J. Fish Dis..

[18] Cherif S., Frikha F., Gargouri Y., Miled N. (2008). Fatty acid composition of green crab (*Carcinus mediterraneus*) from the Tunisian mediterranean coasts. Food Chem..

[19] Nguyen T.P.L., Nguyen V.T.A., Do T.T.T., Nguyen Quang T., Pham Q.L., Le T.T. (2020). Fatty Acid Composition, Phospholipid Molecules, and Bioactivities of Lipids of the Mud Crab *Scylla paramamosain*. J. Chem..

[20] Zhang J., Wu X., Qiu J., Zhang L., Zhang Y., Qiu X., Huang Z., Xu W. (2020). Comprehensive Comparison on the Chemical Profile of Guang Chen Pi at Different Ripeness Stages Using Untargeted and Pseudotargeted Metabolomics. J. Agric. Food Chem..

[21] Dvoretsky A.G., Bichkaeva F.A., Baranova N.F., Dvoretsky V.G. (2021). Fatty acid composition of the Barents Sea red king crab (*Paralithodes camtschaticus*) leg meat. J. Food Compost. Anal..

[22] Wang T., Xiao X., Regenstein J.M., Wu W., Zhou Y., Wang S., Cheng Y., Wu X., Bao B. (2019). Effect on lipid metabolism of mice continuously fed a crab-containing diet. Food Biosci..

[23] Stoner A.W., Ottmar M.L., Copeman L.A. (2010). Temperature effects on the molting, growth, and lipid composition of newly-settled red king crab. J. Exp. Mar. Biol. Ecol..

[24] Wu H., Ge M., Zhou X., Jiang S., Lin L., Lu J. (2019). Nutritional qualities of normal and precocious adult male Chinese mitten crabs (*Eriocheir sinensis*). Aquac. Res..

[25] Han T., Wang J., Hu S., Li X., Jiang Y., Wang C. (2015). Effects of different dietary lipid sources on growth performance and tissue fatty acid composition of juvenile swimming crab *Portunus trituberculatus*. Chin. J. Oceanol. Limnol..

[26] Liu F., Ren D., Guo D.A., Pan Y., Zhang H., Hu P. (2008). Method Development for Gypenosides Fingerprint by High Performance Liquid Chromatography with Diode-Array Detection and the Addition of Internal Standard. Chem. Pharm. Bull..

[27] Huang C., Li Y., Wang K., Xi J., Xu Y., Si X., Pei D., Lyu S., Xia G., Wang J. (2022). Analysis of lipidomics profile of Carya cathayensis nuts and lipid dynamic changes during embryonic development. Food Chem..

[28] Wang F., Lin W., Lv S., Jiang S., Lin L., Lu J. (2019). Comparison of Lipids Extracted by Different Methods from Chinese Mitten Crab (*Eriocheir sinensis*) Hepatopancreas. J. Food Sci..

[29] Jobling M., Johansen S., Foshaug H., Burkow I.C., JøRgensen E.H. (1998). Lipid dynamics in anadromous Arctic charr, Salvelinus alpinus (L.): Seasonal variations in lipid storage depots and lipid class composition. Fish Physiol. Biochem..

[30] Wang W., Wu X., Liu Z., Zheng H., Cheng Y. (2014). Insights into hepatopancreatic functions for nutrition metabolism and ovarian development in the crab *Portunus trituberculatus*: Gene discovery in the comparative transcriptome of different hepatopancreas stages. PLoS ONE.

[31] Lu Z., Shi C., Liu L., Mu C., Ye Y., Wang C. (2022). Phospholipid Compositions in *Portunus trituberculatus* Larvae at Different Developmental Stages. J. Ocean. Univ. China.

[32] Zhang Y., Zhang M., Dong L., Chang J., Wang H., Shen Q. (2021). Lipidomics Screening of Polyunsaturated Phospholipid Molecular Species in Crab (*Portunus trituberculatus*) Muscular Tissue: A Nontarget Approach by HILIC-MS. Eur. J. Lipid Sci. Technol..

[33] Suprayudi M.A., Takeuchi T., Hamasaki K. (2012). Phospholipids Effect on Survival and Molting Synchronicity of Larvae Mud Crab Scylla serrata. HAYATI J. Biosci..

[34] Xu H., Wang J., Han T., Li X., Zheng P., Yin F., Wang C. (2018). Effects of dietary phospholipids levels on growth performance, lipid metabolism, and antioxidant capacity of the early juvenile green mud crab, *Scylla paramamosain* (Estampador). Aquac. Res..

[35] Fitzpatrick J. (1982). Subjectivity and objectivity: Polanyi and Lonergan. High. Educ. Q..

[36] Weiser M.J., Butt C.M., Mohajeri M.H. (2016). Docosahexaenoic Acid and Cognition throughout the Lifespan. Nutrients.

[37] Hossain Z., Hosokawa M., Takahashi K. (2009). Growth inhibition and induction of apoptosis of colon cancer cell lines by applying marine phospholipid. Nutr. Cancer.

[38] Zhang L.Y., Ding L., Shi H.H., Xu J., Xue C.H., Zhang T.T., Wang Y.M. (2019). Eicosapentaenoic acid in the form of phospholipids exerts superior anti-atherosclerosis effects to its triglyceride form in ApoE(-/-) mice. Food Funct..

[39] Zhang T.T., Xu J., Wang Y.M., Xue C.H. (2019). Health benefits of dietary marine DHA/EPA-enriched glycerophospholipids. Prog. Lipid Res..

[40] Donovan E.L., Pettine S.M., Hickey M.S., Hamilton K.L., Miller B.F. (2013). Lipidomic analysis of human plasma reveals ether-linked lipids that are elevated in morbidly obese humans compared to lean. Diabetol. Metab. Syndr..

[41] Rangholia N., Leisner T.M., Holly S.P. (2021). Bioactive Ether Lipids: Primordial Modulators of Cellular Signaling. Metabolites.

[42] Aboshi T., Nishida R., Mori N. (2012). Identification of plasmalogen in the gut of silkworm (*Bombyx mori*). Insect Biochem. Mol. Biol..

[43] Zemski Berry K.A., Murphy R.C. (2004). Electrospray ionization tandem mass spectrometry of glycerophosphoethanolamine plasmalogen phospholipids. J. Am. Soc. Mass. Spectrom..

[44] Liu Z.Y., Zhou D.Y., Wu Z.X., Yin F.W., Zhao Q., Xie H.K., Zhang J.R., Qin L., Shahidi F. (2018). Extraction and detailed characterization of phospholipid-enriched oils from six species of edible clams. Food Chem..

[45] Wiklund S., Johansson E., Sjöström L., Mellerowicz E.J., Edlund U., Shockcor J.P., Gottfries J., Moritz T., Trygg J. (2008). Visualization of GC/TOF-MS-Based Metabolomics Data for Identification of Biochemically Interesting Compounds Using OPLS Class Models. Anal. Chem..

[46] Liu H., Hui T., Zheng X., Li S., Wei X., Li P., Zhang D., Wang Z. (2022). Characterization of key lipids for binding and generating aroma compounds in roasted mutton by UPLC-ESI-MS/MS and Orbitrap Exploris GC. Food Chem..

[47] He C., Cao J., Bao Y., Sun Z., Liu Z., Li C. (2021). Characterization of lipid profiling in three parts (muscle, head and viscera) of tilapia (*Oreochromis niloticus*) using lipidomics with UPLC-ESI-Q-TOF-MS. Food Chem..

[48] Roth B., Ines S. (2010). Stunning and killing of edible crabs (*Cancer pagurus*). Anim. Welf..

[49] Yang F., Zhao M., Zhou L., Zhang M., Liu J., Marchioni E. (2021). Identification and Differentiation of Wide Edible Mushrooms Based on Lipidomics Profiling Combined with Principal Component Analysis. J. Agric. Food Chem..

[50] Song S., Cheong L.Z., Wang H., Man Q.Q., Pang S.J., Li Y.Q., Ren B., Wang Z., Zhang J. (2018). Characterization of phospholipid profiles in six kinds of nut using HILIC-ESI-IT-TOF-MS system. Food Chem..

[51] Sun F., Chen H., Chen D., Tan H., Huang Y., Cozzolino D. (2020). Lipidomic Changes in Banana (*Musa cavendish*) during Ripening and Comparison of Extraction by Folch and Bligh-Dyer Methods. J. Agric. Food Chem..

[52] Shi C., Guo H., Wu T., Tao N., Wang X., Zhong J. (2019). Effect of three types of thermal processing methods on the lipidomics profile of tilapia fillets by UPLC-Q-Extractive Orbitrap mass spectrometry. Food Chem..

