# Supplementary material for: Untargeted Lipidomics Method for the Discrimination of Five Crab Species by Ultra-High-Performance Liquid Chromatography High-Resolution Mass Spectrometry Combined with Chemometrics
Source: Molecules. 2023 Apr 22;28(9):3653. doi: 10.3390/molecules28093653 (PMC10179896; doi:10.3390/molecules28093653)
Supplement: Supplementary file 1 [file molecules-28-03653-s001.zip › molecules-2267932-supplementary.pdf]

Supplementary materials

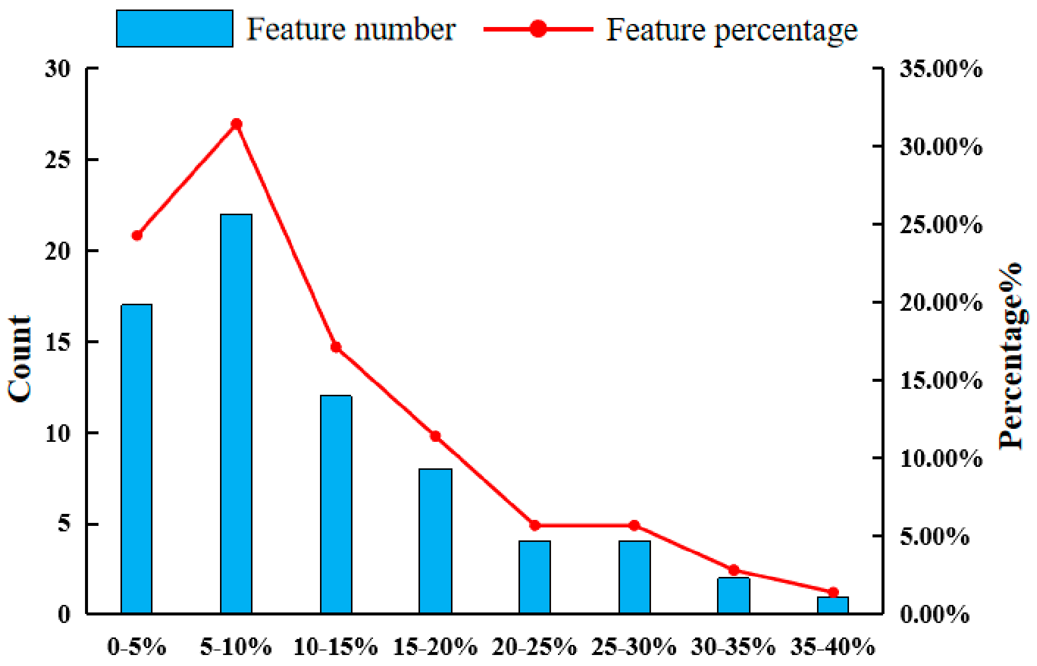

Figure S1. RSD distribution of peak area of seven internal standards in 10 groups of different samples.

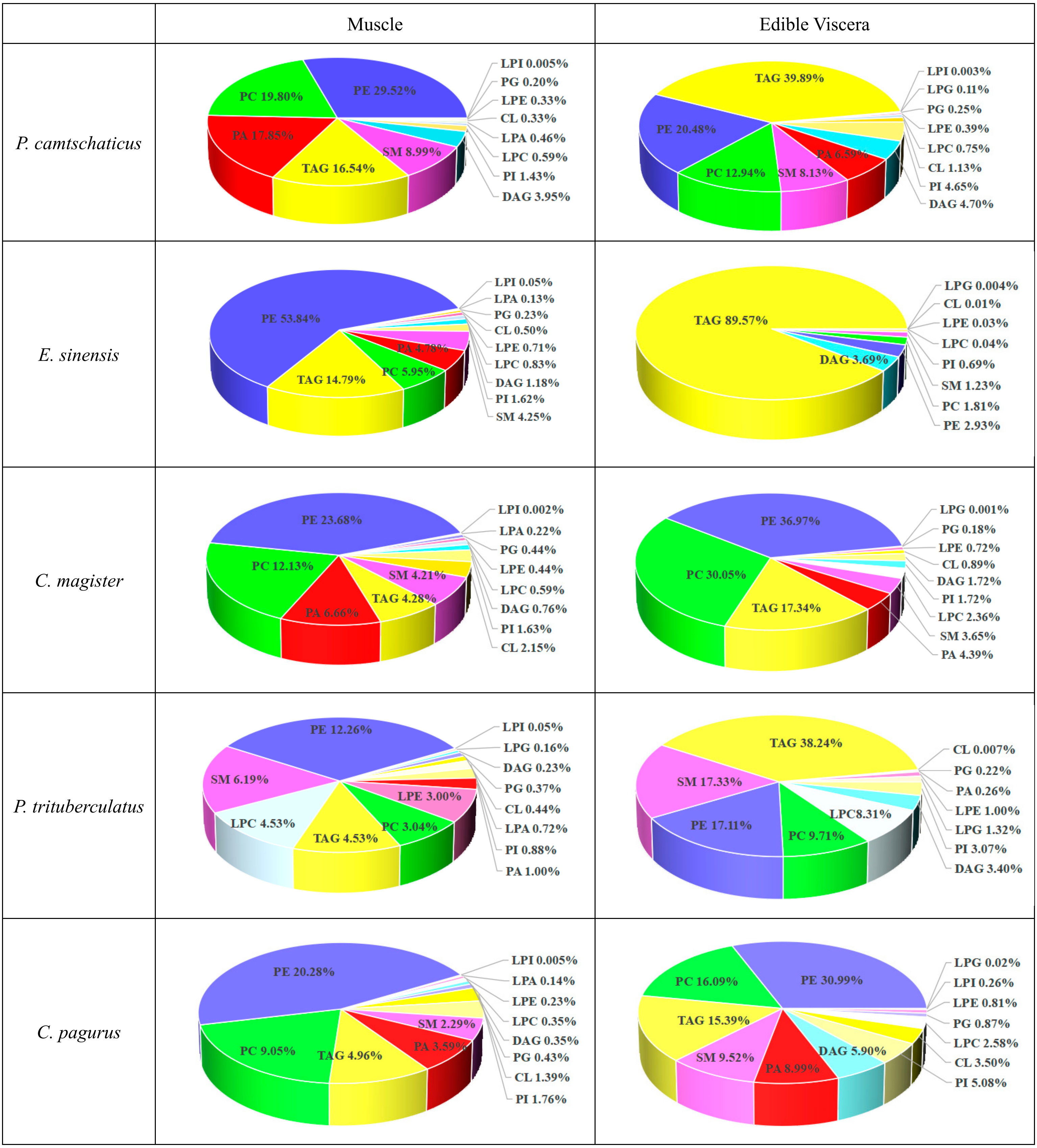

Figure S2. Lipid composition (%) in muscles and edible viscera of five crab species

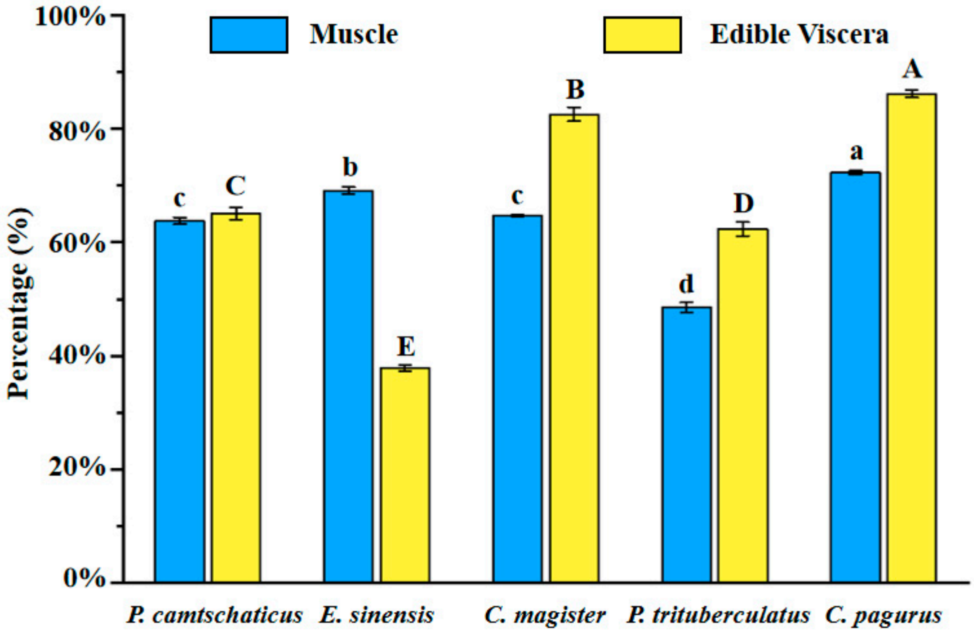

Figure S3. The percentage contents of DHA/EPA-PLs and DHA/EPA-TAGs in total lipid extracted from crab muscle and edible viscera. Data are expressed as the mean ± SD. Different letters indicate significant difference ($p < 0.05$) among different samples.

(a)

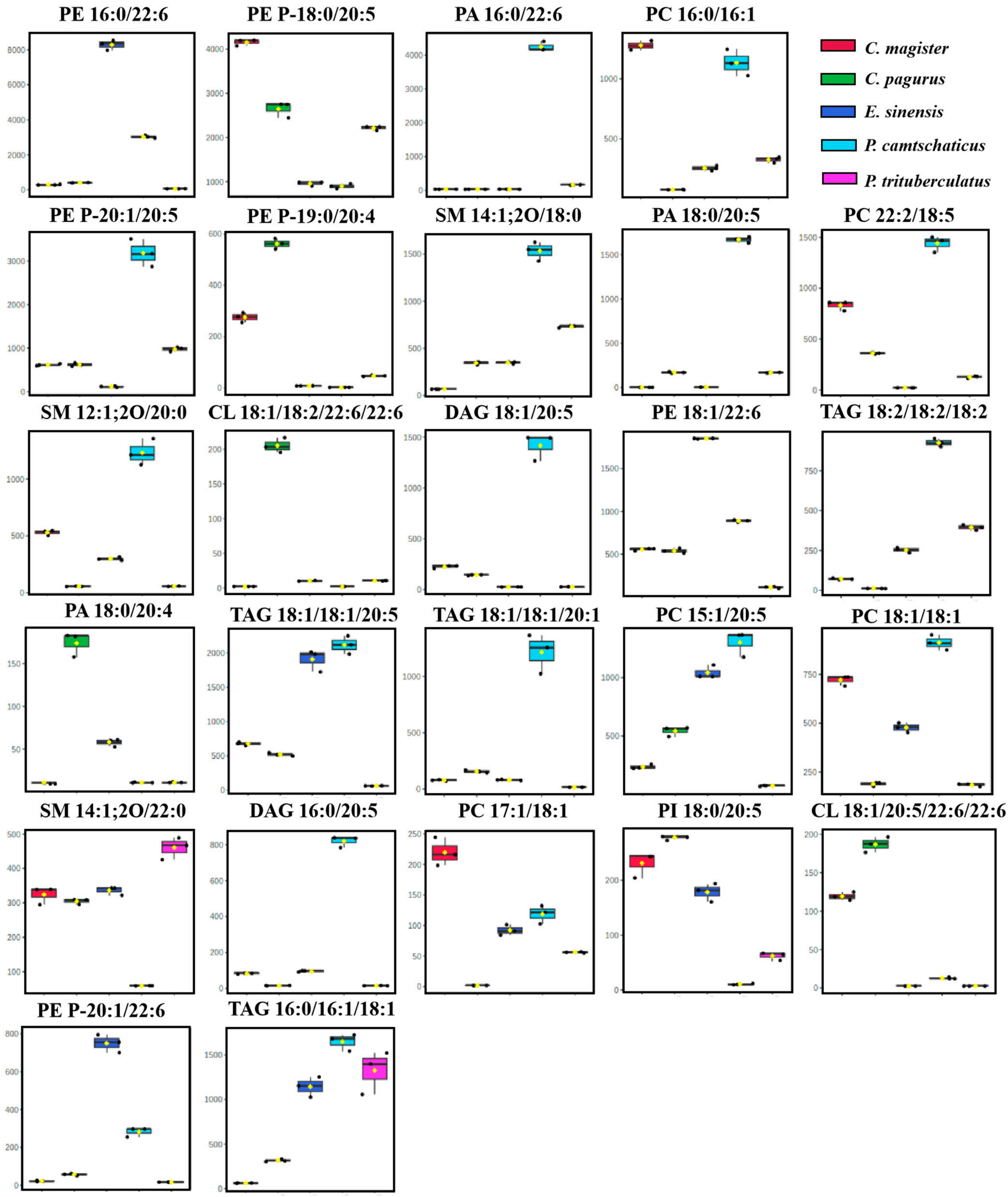

PE 16:0/22:6
PE P-18:0/20:5
PA 16:0/22:6
PC 16:0/16:1
C. magister
C. pagurus
E. sinensis
P. camtschaticus
P. trituberculatus
PE P-20:1/20:5
PE P-19:0/20:4
SM 14:1;2O/18:0
PA 18:0/20:5
PC 22:2/18:5
SM 12:1;2O/20:0
CL 18:1/18:2/22:6/22:6
DAG 18:1/20:5
PE 18:1/22:6
TAG 18:2/18:2/18:2
PA 18:0/20:4
TAG 18:1/18:1/20:5
TAG 18:1/18:1/20:1
PC 15:1/20:5
PC 18:1/18:1
SM 14:1;2O/22:0
DAG 16:0/20:5
PC 17:1/18:1
PI 18:0/20:5
CL 18:1/20:5/22:6/22:6
PE P-20:1/22:6
TAG 16:0/16:1/18:1

(b)

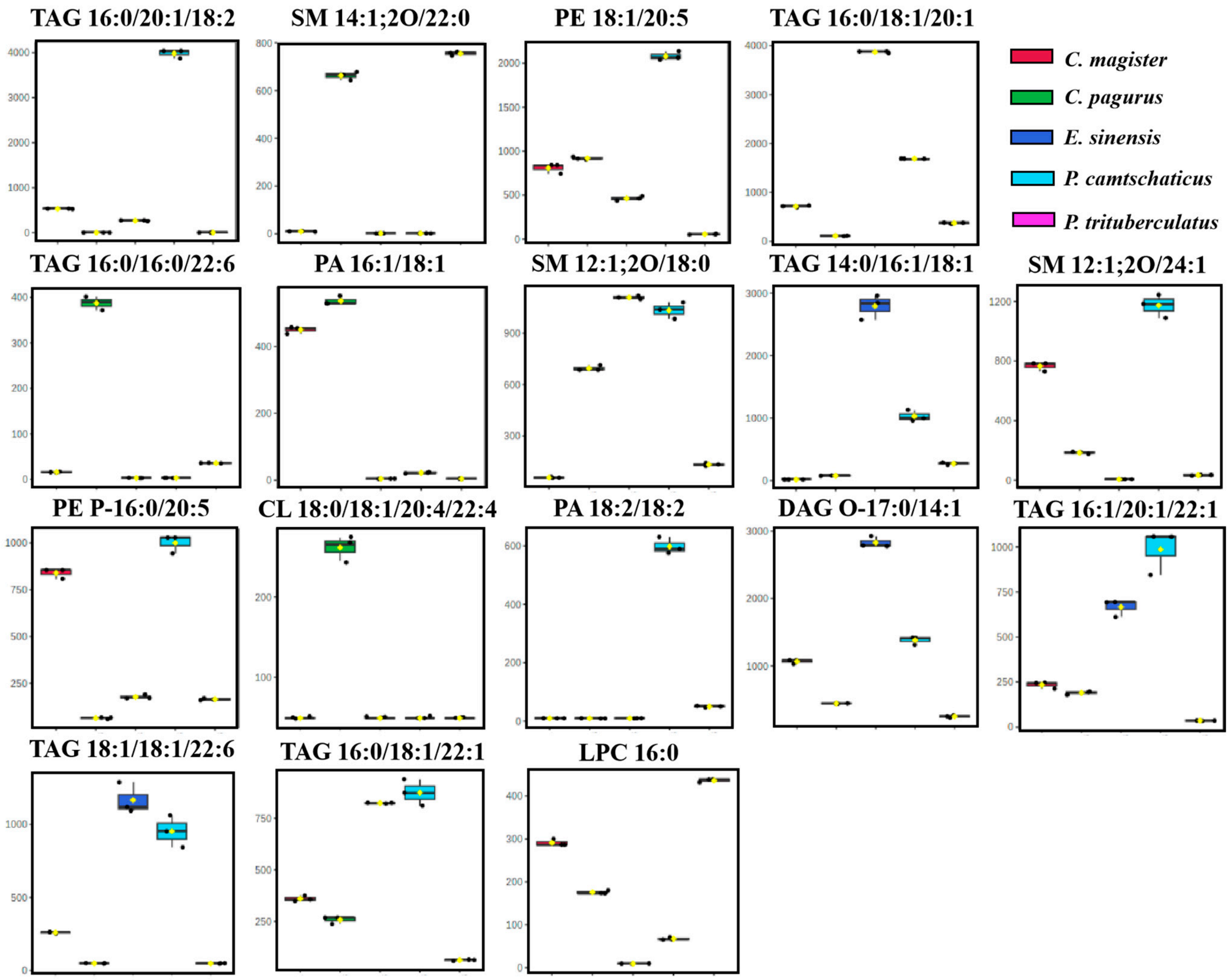

Figure S4. Box plots of the significantly different lipids in muscles (a) and edible viscera (b) of five crab species

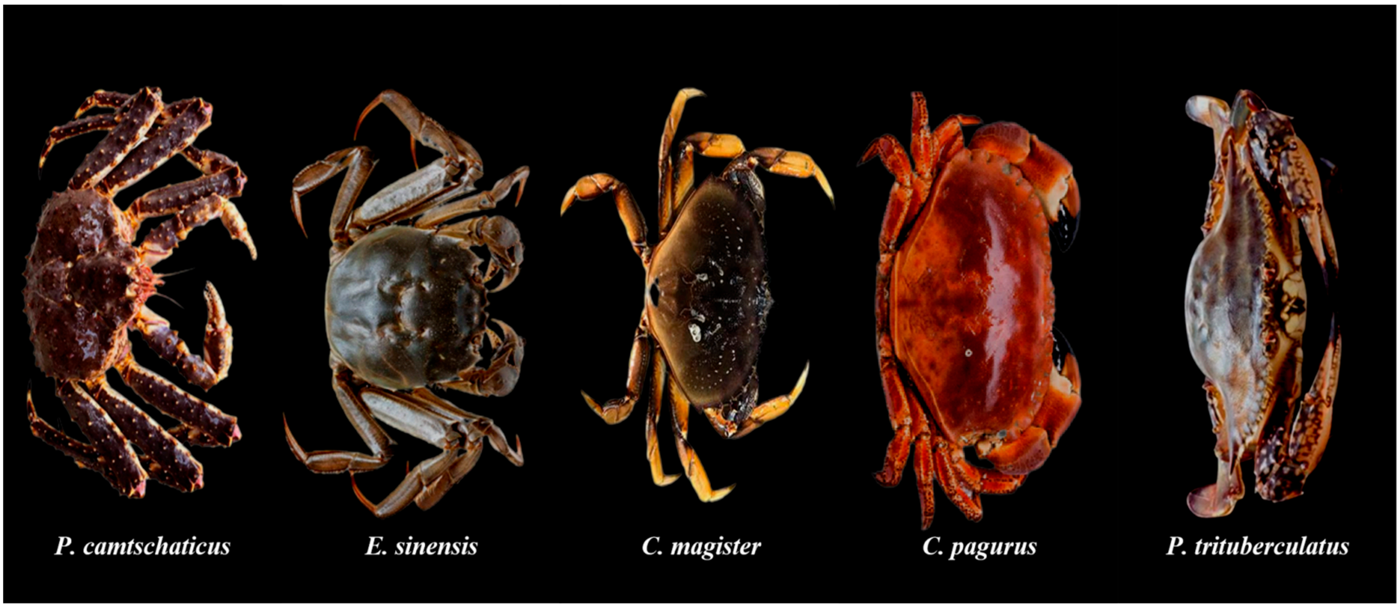

Figure S5: Optical images of the crab species.

Table S1. Concentration (ng/mL) of 7 lipid standards and corresponding internal standard (IS) for construction of calibration curves

Standard	Conc. 1	Conc. 2	Conc. 3	Conc. 4	Conc. 5	Conc. 6	Conc. 7	IS	IS Conc.
PI 8:0/8:0	62500	12500	6250	1250	625	312	125	PI 15:0/18:1(d7)	1666
PE 17:0/17:0	433500	86700	43350	8670	4335	2168	867	PE 15:0/18:1(d7)	833
PC 17:0/17:0	562500	112500	56250	11250	5625	2812	1125	PC 15:0/18:1(d7)	26666
PG 17:0/17:0	546000	109200	54600	10920	5460	2730	1092	PG 15:0/18:1(d7)	5000
PA 17:0/17:0	521000	104200	52100	10420	5210	2605	1042	PA 15:0/18:1(d7)	1166
SM d18:1/12:0	866500	173300	86650	17330	8665	4332	1733	SM d18:1/18:1(d9)	5000
TAG 18:2/18:2/18:2	5700000	1140000	570000	114000	57000	28500	11400	TAG 15:0/18:1(d7)/15:0	9166

Table S2. Total lipids content in five kinds of crabs

Species	Muscle	Edible viscera
	mg/g (dry powder)	mg/g (dry powder)
P. camtschaticus	193.3±15.4[a]	766.3±35.1[a]
E. sinensis	157.6±17.8[a]	761.0±111.8[a]
C. magister	197.6±20.4[a]	426.7±11.5[c]
P. trituberculatus	182.8±32.0[a]	594.4±4.9[b]
C. pagurus	196.7±40.4[a]	516.2±12.2[bc]

Data are expressed as the mean ± SD. Different letters in the same column indicate significant difference ($p < 0.05$) among different samples.

Table S3. The linear regression equations, linear regression coefficients (R^2), limit of detection (LOD) and limit of quantification (LOQ) of lipid standards

Standards	Internal Standard	Equations	R^2	LOD (ng/mL)	LOQ (ng/mL)
PC 17:0/17:0	PC 15:0/18:1(d7)	y=15.37x+0.01	0.996	2.09	6.89
PG 17:0/17:0	PG 15:0/18:1(d7)	y=106.5x-0.1	0.997	1.19	3.93
PE 17:0/17:0	PE 15:0/18:1(d7)	y=105.1x+0.4	0.995	0.30	0.99
TAG 18:2/18:2/18:2	TAG 15:0/18:1(d7)/15:0	y=0.92x+0.01	0.999	2.28	7.52
PI 8:0/8:0	PI 15:0/18:1(d7)	y=1313.7x-1.5	0.998	1.76	5.80
SM d18:1/12:0	SM d18:1/18:1(d9)	y=142.8x-1.3	0.999	0.34	1.14
PA 17:0/17:0	PA 15:0/18:1(d7)	y=434.4x-3.0	0.996	1.60	5.27

x means the concentration (mg/mL) of lipid, y means the ratio of the peak area of lipid to the peak area of the internal standard.

Table S4. Molecular species of lipids extracted from muscles of five kinds of crabs

Type	Species	*m/z*	*m/z* ref	ppm	Concentrations (nmol/g)				
					P. camtschaticus	*E. sinensis*	*C. magister*	*P. trituberculatus*	*C. pagurus*
					LPC				
$[M+H]^+$	16:0	496.3398	496.3398	0.00	147.2±12.6	117.8±4.57	85.69±2.64	83.09±4.25	44.77±4.32
$[M+H]^+$	16:1	494.3242	494.3241	0.20	10.27±1.08	28.89±3.58	23.82±1.47	205.6±3.87	6.52±0.37
$[M+H]^+$	18:1	522.3553	522.3554	-0.19	177.6±10.1	223.5±10.3	142.5±7.1	1471±137	103.1±5.1
$[M+H]^+$	19:0	538.3873	538.3867	1.11	nd	nd	1.94±0.10	57.64±2.64	nd
$[M+H]^+$	20:1	550.3868	550.3867	0.18	11.34±0.00	2.29±0.18	nd	30.24±1.65	nd
$[M+H]^+$	20:2	548.3710	548.3711	-0.18	nd	4.79±0.23	9.67±0.68	31.22±1.89	nd
$[M+H]^+$	20:5	542.3245	542.3241	0.74	110.7±2.2	60.65±5.68	79.76±7.04	504.1±40.4	60.25±5.88
$[M+H]^+$	21:2	584.3712	584.3687	4.28	nd	nd	6.84±0.35	49.11±4.58	2.14±0.18
$[M+H]^+$	21:5	556.3396	556.3398	-0.36	nd	1.10±0.09	1.87±0.10	19.32±1.79	1.57±0.07
$[M+H]^+$	22:6	568.3391	568.3398	-1.23	61.78±1.40	149.5±14.2	42.80±3.67	481.3±48.2	28.20±2.35
$[M+H]^+$	O-15:0	468.3456	468.3449	1.49	1.47±0.10	2.06±0.05	nd	145.7±11.4	nd
$[M+H]^+$	O-16:1	480.3448	480.3449	-0.21	9.01±1.15	nd	17.34±1.13	47.88±1.73	3.28±0.26
$[M+H]^+$	O-16:4	474.2990	474.2979	2.32	nd	1.64±0.10	1.19±0.15	11.13±0.53	nd
$[M+H]^+$	O-18:1	508.3768	508.3762	1.18	nd	5.25±0.26	11.26±0.84	120.3±12.5	1.23±0.13
$[M+H]^+$	O-20:2	534.3928	534.3918	1.87	1.36±0.12	nd	nd	3.36±0.38	nd
					PC				
$[M+H]^+$	15:1/20:5	764.5219	764.5225	-0.78	1304±108	1043±56	233.5±18.3	71.81±3.80	542.3±42.7
$[M+H]^+$	16:0/16:1	732.5544	732.5538	0.82	1134±111	255.7±21.6	1279±40	326.4±23.5	76.55±2.79
$[M+H]^+$	16:0/20:5	780.5530	780.5538	-1.02	2491±156	296.6±22.0	910.6±33.5	242.1±22.5	1325±68
$[M+H]^+$	16:1/20:5	778.5377	778.5381	-0.51	2.07±0.08	75.32±6.57	276.6±22.7	81.89±4.00	54.86±5.50
$[M+H]^+$	16:4/22:5	800.5221	800.5225	-0.50	104.2±9.6	nd	51.57±3.09	nd	nd
$[M+H]^+$	17:1/18:1	772.5858	772.5851	0.91	118.6±15.1	92.12±8.64	219.6±23.2	55.86±1.08	1.58±0.09
$[M+H]^+$	17:1/22:6	818.5684	818.5694	-1.22	277.3±12.0	37.62±4.62	131.9±11.4	74.15±4.00	55.93±5.06
$[M+H]^+$	18:0/20:2	814.6309	814.6320	-1.35	210.5±1.48	nd	nd	nd	nd
$[M+H]^+$	18:1/18:1	786.6015	786.6007	1.02	912.8±38.6	477.7±25.1	720.9±25.8	183.6±7.3	188.3±10.2
$[M+H]^+$	20:5/20:5	826.5369	826.5381	-1.45	684.3±8.7	nd	392.0±18.6	98.51±7.65	878.9±75.1
$[M+H]^+$	20:5/21:5	840.5532	840.5538	-0.71	45.16±4.96	6.14±0.17	30.87±2.51	nd	54.93±2.43
$[M+H]^+$	20:5/22:6	852.5530	852.5538	-0.94	622.8±22.5	242.1±16.8	505.1±16.3	152.7±15.8	590.7±17.2

$[M+H]^+$	21:5/22:6	866.5689	866.5694	-0.58	24.57±0.32	9.86±0.78	18.83±1.46	3.88±0.12	18.64±1.58
$[M+H]^+$	22:2/18:5	832.5856	832.5851	0.60	1441±78	nd	832.3±46.9	127.5±12.3	361.9±8.0
$[M+H]^+$	22:6/22:6	878.5695	878.5694	0.11	116.2±5.6	329.7±23.3	220.4±17.8	42.55±3.49	115.6±5.6
					LPE				
$[M+H]^+$	20:5	500.2772	500.2772	0.00	163.5±17.5	212.1±9.8	202.8±0.3	315.7±11.6	143.4±1.4
$[M+H]^+$	22:6	526.2934	526.2928	1.14	33.56±3.05	217.1±15.1	17.17±1.66	528.4±8.5	22.65±2.06
$[M+H]^+$	O-17:1	452.3141	452.3136	1.11	nd	nd	nd	103.4±0.2	nd
$[M+H]^+$	O-18:1	466.3293	466.3292	0.21	52.35±2.02	105.4±13.5	123.4±11.5	1412±38	7.66±0.42
					PE				
$[M+H]^+$	16:0/16:1	690.5065	690.5068	-0.43	218.7±3.8	308.7±15.4	175.0±7.6	49.60±4.06	43.60±2.99
$[M+H]^+$	16:0/18:1	718.5386	718.5381	0.70	163.1±5.6	1260±82	105.3±2.7	55.56±1.14	21.49±2.04
$[M+H]^+$	16:0/18:2	716.5221	716.5225	-0.56	71.76±3.88	nd	19.97±1.56	30.52±2.97	21.34±1.87
$[M+H]^+$	16:0/22:6	764.5239	764.5225	1.83	3034±80	8278±285	296.6±22.2	nd	404.6±14.7
$[M+H]^+$	16:1/18:1	716.5225	716.5225	0.00	103.8±6.0	872.8±6.9	nd	nd	nd
$[M+H]^+$	17:0/18:2	730.5381	730.5381	0.00	384.4±18.6	45.82±3.40	513.0±16.2	14.39±0.93	183.8±5.1
$[M+H]^+$	18:0/18:2	744.5544	744.5538	0.81	134.6±10.1	706.9±19.1	180.7±19.1	36.84±1.47	84.62±5.16
$[M+H]^+$	18:0/18:5	738.5065	738.5068	-0.41	1006±60	729.7±4.1	605.7±37.8	33.51±1.48	927.1±8.3
$[M+H]^+$	18:0/20:5	766.5386	766.5381	0.65	13.25±0.95	4968±144	1589±104	215.7±9.5	298.5±22.5
$[M+H]^+$	18:1/22:6	790.5383	790.5381	0.25	889.3±14.1	1852±4	556.2±14.5	113.3±11.6	540.1±29.6
$[M+H]^+$	22:6/22:6	836.5208	836.5225	-2.03	69.66±5.49	1238±104	167.0±8.0	52.14±2.41	124.6±2.4
$[M+H]^+$	P-16:0/16:1	690.5079	690.5068	1.59	11.20±1.04	nd	42.67±2.58	42.19±3.67	nd
$[M+H]^+$	P-16:0/18:1	702.5435	702.5432	0.43	85.26±6.40	262.8±13.7	42.81±2.50	71.73±3.64	3.77±0.45
$[M+H]^+$	P-16:0/20:5	722.5123	722.5119	0.55	365.6±34.3	323.8±8.6	419.2±18.4	181.4±20.4	371.4±7.3
$[M+H]^+$	P-16:0/22:6	748.5275	748.5276	-0.13	2609±154	755.8±58.5	1147±22	663.0±9.7	1531±43
$[M+H]^+$	P-17:0/20:5	736.5276	736.5276	0.00	133.1±4.6	379.9±18.1	292.3±18.1	155.4±12.4	122.9±11.0
$[M+H]^+$	P-17:0/22:6	762.5427	762.5432	-0.66	213.2±12.2	1520±125	325.2±27.9	214.6±3.2	137.9±10.9
$[M+H]^+$	P-18:0/20:5	750.5430	750.5432	-0.27	897.5±56.9	956.4±46.8	4156±70	2216±47	2649±175
$[M+H]^+$	P-18:0/22:6	776.5593	776.5589	0.52	250.9±7.4	1421±82	295.6±9.9	823.7±51.0	997.4±19.5
$[M+H]^+$	P-18:1/22:6	774.5424	774.5432	-1.03	642.8±56.1	47.92±4.31	84.03±7.26	133.4±11.5	462.7±39.8
$[M+H]^+$	P-19:0/20:4	766.5734	766.5745	-1.43	nd	8.14±0.27	275.5±18.9	47.09±2.60	561.9±20.7
$[M+H]^+$	P-20:1/20:5	776.5576	776.5589	-1.67	3183±317	116.5±14.0	615.4±24.6	977.4±52.4	620.8±39.2
$[M+H]^+$	P-20:1/22:6	802.5745	802.5745	0.00	283.0±24.7	750.4±47.7	22.48±2.76	16.43±0.80	56.83±6.10
					TAG				

$[M+NH_4]^+$	14:0/16:0/18:1	822.7538	822.7545	-0.85	nd	624.2±29.6	nd	nd	95.59±6.43
$[M+NH_4]^+$	16:0/16:1/18:1	848.7702	848.7678	2.83	1650±95	1144±114	61.29±3.62	1326±241	315.3±15.3
$[M+NH_4]^+$	16:0/16:1/20:1	876.7996	876.8015	-2.17	584.0±57.4	749.1±25.5	197.5±8.9	nd	nd
$[M+NH_4]^+$	16:0/18:1/18:1	876.7993	876.8015	-2.51	520.9±20.5	348.0±42.3	541.9±8.6	102.7±19.9	1041±58
$[M+NH_4]^+$	16:0/18:1/20:1	904.8304	904.8328	-2.65	nd	1227±153	183.4±14.6	102.7±4.5	nd
$[M+NH_4]^+$	18:1/18:1/20:1	930.8484	930.8475	0.96	1215±174	80.37±5.06	76.26±6.06	nd	154.8±13.0
$[M+NH_4]^+$	18:1/18:1/20:5	922.7850	922.7858	-0.87	2119±132	1909±159	677.8±25.0	62.41±2.85	520.9±25.6
$[M+NH_4]^+$	18:2/18:2/18:2	896.7681	896.7702	-2.34	924.8±25.1	252.8±15.4	71.45±7.06	394.7±18.6	nd
					DAG				
$[M+NH_4]^+$	16:0/20:5	632.5250	632.5249	0.16	819.2±30.0	94.78±4.29	83.02±3.21	nd	nd
$[M+NH_4]^+$	16:0/22:6	658.5405	658.5405	0.00	nd	476.5±16.4	nd	nd	nd
$[M+NH_4]^+$	18:1/20:5	658.5404	658.5405	-0.15	1415±131	nd	225.9±18.7	nd	144.3±5.6
$[M+NH_4]^+$	O-15:0/14:1	528.4987	528.4986	0.19	23.35±0.51	11.71±0.39	2.56±0.18	2.13±0.30	nd
$[M+NH_4]^+$	O-15:0/16:1	556.5297	556.5299	-0.36	68.22±4.76	nd	17.11±0.56	nd	nd
$[M+NH_4]^+$	O-17:0/14:1	556.5299	556.5299	0.00	nd	126.2±6.8	137.8±11.9	152.6±23.9	68.61±0.84
					SM				
$[M+HCOO]^-$	12:1;2O/18:0	691.5034	691.5032	0.29	308.5±14.6	29.49±2.92	42.42±3.46	228.6±5.6	20.93±2.50
$[M+HCOO]^-$	12:1;2O/20:0	719.5349	719.5345	0.56	1231±117	299.2±12.6	530.1±22.9	nd	nd
$[M+HCOO]^-$	12:1;2O/24:1	773.5820	773.5814	0.78	588.0±41.8	6.83±0.57	153.7±7.7	55.86±5.56	94.47±0.85
$[M+HCOO]^-$	14:1;2O/18:0	719.5351	719.5345	0.83	1533±100	347.6±13.3	nd	729.8±12.3	343.6±16.6
$[M+HCOO]^-$	14:1;2O/19:0	733.5509	733.5501	1.09	326.1±19.7	249.9±5.9	328.3±21.7	407.3±14.9	45.46±2.71
$[M+HCOO]^-$	14:1;2O/20:0	747.5664	747.5658	0.80	388.0±36.3	395.4±4.7	449.0±0.4	576.2±32.9	316.7±6.6
$[M+HCOO]^-$	14:1;2O/21:0	761.5818	761.5814	0.53	nd	269.4±26.7	269.8±16.8	253.9±9.7	14.26±1.00
$[M+HCOO]^-$	14:1;2O/22:0	775.5978	775.5971	0.90	nd	336.2±12.3	324.2±25.4	460.6±32.5	304.8±7.8
$[M+HCOO]^-$	14:1;2O/23:0	789.6135	789.6127	1.01	nd	221.5±20.8	27.62±1.88	92.65±3.69	12.23±0.81
$[M+HCOO]^-$	14:1;2O/24:1	801.6134	801.6127	0.87	287.6±1.9	3.71±0.39	17.42±0.73	30.09±2.87	10.20±0.66
$[M+HCOO]^-$	16:1;2O/20:0	775.5980	775.5971	1.16	nd	nd	nd	318.8±15.4	nd
					CL				
$[M-2H]^{2-}$	16:0/18:1/18:2/20:4	724.4863	724.4867	-0.55	13.45±1.17	nd	nd	8.46±0.47	12.93±0.97
$[M-2H]^{2-}$	16:1/16:1/16:1/18:1	685.4632	685.4630	0.29	nd	nd	3.23±0.22	11.57±0.39	nd
$[M-2H]^{2-}$	16:1/16:1/16:1/20:5	695.4482	695.4475	1.01	nd	nd	13.22±0.41	2.29±0.16	nd
$[M-2H]^{2-}$	16:1/16:1/18:1/20:5	709.4633	709.4632	0.14	13.54±1.08	27.57±1.31	55.9±5.16	22.19±2.38	2.45±0.27
$[M-2H]^{2-}$	16:1/16:1/18:2/20:5	708.4554	708.4554	0.00	nd	19.46±1.10	14.02±0.17	22.29±2.00	3.31±0.13

$[M-2H]^{2-}$	16:1/16:1/20:5/20:5	719.4473	719.4475	-0.28	5.09±0.13	4.23±0.29	13.41±0.37	nd	nd
$[M-2H]^{2-}$	16:1/16:1/20:5/22:6	732.4534	732.4554	-2.73	nd	nd	nd	4.77±0.50	12.17±1.10
$[M-2H]^{2-}$	16:1/17:1/22:6/22:6	752.4707	752.4710	-0.40	nd	nd	14.12±1.16	nd	9.66±0.51
$[M-2H]^{2-}$	16:1/18:1/18:1/18:2	712.4865	712.4867	-0.28	2.29±0.16	nd	14.71±1.08	13.40±1.24	8.61±0.14
$[M-2H]^{2-}$	16:1/18:1/18:2/20:5	722.4714	722.4710	0.55	14.20±1.00	nd	114.4±11.5	nd	13.14±0.80
$[M-2H]^{2-}$	16:1/18:1/20:3/20:5	735.4799	735.4788	1.50	15.41±0.73	89.34±3.80	103.7±8.4	nd	nd
$[M-2H]^{2-}$	16:1/18:2/18:2/20:5	721.4626	721.4632	-0.83	13.17±1.09	5.04±0.19	15.68±1.45	nd	2.52±0.05
$[M-2H]^{2-}$	16:1/18:2/20:5/20:5	732.4553	732.4554	-0.14	4.46±0.09	8.22±0.31	16.61±0.11	nd	nd
$[M-2H]^{2-}$	16:1/18:2/20:5/22:6	745.4640	745.4630	1.34	7.51±0.43	14.44±1.01	nd	10.91±0.37	nd
$[M-2H]^{2-}$	16:1/18:2/22:6/22:6	758.4707	758.4710	-0.40	8.72±0.23	16.47±0.78	nd	10.74±0.36	nd
$[M-2H]^{2-}$	16:1/20:4/20:5/20:5	744.4543	744.4554	-1.48	1.46±0.10	nd	14.55±1.29	nd	6.177±0.420
$[M-2H]^{2-}$	16:1/20:5/20:5/20:5	743.4478	743.4475	0.40	1.06±0.05	nd	13.77±1.17	2.47±0.32	1.32±0.05
$[M-2H]^{2-}$	16:1/20:5/20:5/22:6	756.4555	756.4554	0.13	12.40±1.00	nd	15.98±1.45	nd	71.37±4.36
$[M-2H]^{2-}$	16:1/20:5/22:6/22:6	769.4634	769.4632	0.26	7.05±0.17	nd	16.43±0.36	nd	12.06±0.47
$[M-2H]^{2-}$	16:1/22:6/22:6/22:6	782.4715	782.4710	0.64	2.81±0.19	nd	16.67±1.16	nd	18.25±1.42
$[M-2H]^{2-}$	18:0/18:1/20:3/22:6	763.5120	763.5101	2.49	nd	24.92±0.29	233.0±15.3	42.69±3.02	nd
$[M-2H]^{2-}$	18:1/18:2/22:6/22:6	772.4867	772.4867	0.00	nd	10.23±0.60	nd	10.32±0.35	205.1±10.7
$[M-2H]^{2-}$	18:1/20:5/22:6/22:6	783.4789	783.4788	0.13	13.01±1.19	nd	119.2±5.2	nd	186.6±10.0
$[M-2H]^{2-}$	18:2/18:2/18:3/20:3	735.4791	735.4788	0.41	14.55±1.02	16.4±0.11	nd	58.47±3.57	nd
$[M-2H]^{2-}$	18:2/18:3/18:3/18:3	720.4562	720.4554	1.11	nd	11.71±0.75	nd	6.66±0.21	2.21±0.13
$[M-2H]^{2-}$	18:2/18:3/18:3/20:5	732.4560	732.4554	0.82	nd	10.51±0.96	nd	nd	nd
$[M-2H]^{2-}$	18:2/18:3/18:3/22:6	745.4635	745.4632	0.40	3.24±0.18	10.53±0.24	132.2±10.0	nd	42.94±2.50
$[M-2H]^{2-}$	18:2/18:3/20:5/20:5	744.4555	744.4554	0.13	nd	10.18±0.94	nd	nd	4.36±0.14
$[M-2H]^{2-}$	18:2/18:3/22:6/22:6	770.4711	770.4710	0.13	9.56±0.30	10.59±0.59	123.6±10.2	3.02±0.15	45.39±3.04
$[M-2H]^{2-}$	18:2/20:5/22:6/22:6	782.4716	782.4710	0.77	4.61±0.27	nd	nd	nd	14.01±1.21
$[M-2H]^{2-}$	18:2/22:6/22:6/22:6	795.4786	795.4788	-0.25	nd	nd	13.16±1.08	nd	11.93±1.14
$[M-2H]^{2-}$	20:5/22:6/22:6/22:6	806.4725	806.4710	1.86	nd	nd	11.89±1.17	nd	1.56±0.05
					LPG				
$[M-H]^-$	16:1	481.2573	481.2572	0.21	nd	nd	nd	17.77±1.12	nd
$[M-H]^-$	18:1	509.2886	509.2885	0.20	nd	nd	nd	20.40±0.79	nd
$[M-H]^-$	18:2	507.2733	507.2728	0.99	nd	nd	nd	15.97±0.46	nd
$[M-H]^-$	20:2	535.3046	535.3041	0.93	nd	nd	nd	17.11±0.51	nd
$[M-H]^-$	20:4	531.2732	531.2728	0.75	nd	nd	nd	15.18±0.14	nd

[M-H]⁻	20:5	529.2571	529.2572	-0.19	nd	nd	nd	15.90±0.56	nd
[M-H]⁻	22:6	555.2730	555.2728	0.36	nd	nd	nd	17.67±0.44	nd
					PG				
[M-H]⁻	16:0/18:1	747.5187	747.5182	0.67	18.19±0.10	13.82±0.08	16.13±1.31	12.41±0.32	13.92±0.85
[M-H]⁻	16:0/19:1	761.5337	761.5338	-0.13	nd	10.09±0.77	12.92±1.08	2.05±0.05	11.37±1.07
[M-H]⁻	16:0/19:2	759.5187	759.5182	0.66	nd	1.38±0.14	2.52±0.03	10.61±0.36	3.74±0.26
[M-H]⁻	16:0/20:2	773.5341	773.5338	0.39	14.28±1.38	27.79±1.33	22.22±1.78	15.89±0.83	26.79±2.59
[M-H]⁻	16:0/20:3	771.5187	771.5182	0.65	1.51±0.14	15.09±0.10	29.93±1.46	13.19±0.65	12.38±0.94
[M-H]⁻	16:0/20:4	769.5029	769.5025	0.52	4.81±0.22	5.28±0.31	14.24±1.06	17.73±1.10	13.28±0.85
[M-H]⁻	16:0/20:5	767.4871	767.4869	0.26	15.09±1.45	3.43±0.14	12.83±1.18	10.51±1.01	13.77±0.84
[M-H]⁻	16:0/22:6	793.5032	793.5025	0.88	15.03±1.26	9.73±0.67	12.80±1.05	nd	12.33±0.91
[M-H]⁻	16:1/16:1	717.4720	717.4712	1.12	nd	nd	1.41±0.04	12.48±0.45	nd
[M-H]⁻	16:1/18:1	745.5027	745.5025	0.27	13.04±1.07	10.94±1.02	13.19±1.07	13.63±0.56	5.57±0.30
[M-H]⁻	16:1/20:5	765.4717	765.4712	0.65	1.21±0.16	nd	11.43±1.05	10.91±0.34	5.14±0.11
[M-H]⁻	16:1/22:6	791.4872	791.4869	0.38	1.62±0.10	nd	12.43±1.01	12.61±0.35	11.04±1.03
[M-H]⁻	17:0/20:2	787.5496	787.5495	0.13	nd	2.13±0.13	12.32±1.04	10.11±0.35	10.92±1.00
[M-H]⁻	17:0/20:5	781.5035	781.5025	1.28	1.23±0.13	nd	nd	nd	11.04±1.07
[M-H]⁻	17:0/22:6	807.5181	807.5182	-0.12	nd	nd	nd	nd	10.56±1.01
[M-H]⁻	18:0/20:5	795.5192	795.5182	1.26	nd	2.31±0.14	22.17±1.63	10.73±0.58	12.19±0.86
[M-H]⁻	18:0/22:6	821.5347	821.5338	1.10	nd	9.34±0.71	2.32±0.16	nd	nd
[M-H]⁻	18:1/22:6	819.5188	819.5182	0.73	12.18±1.19	nd	11.77±0.94	1.15±0.10	11.17±0.94
[M-H]⁻	18:2/22:6	817.5035	817.5025	1.22	nd	nd	2.18±0.17	10.48±0.31	2.87±0.12
[M-H]⁻	20:2/22:6	845.5349	845.5338	1.30	nd	nd	1.89±0.08	9.23±0.30	10.16±0.96
[M-H]⁻	22:6/22:6	865.5035	865.5025	1.16	nd	nd	nd	9.13±0.26	9.94±0.85
					LPA				
[M-H]⁻	16:0	409.2363	409.2361	0.49	138.9±17.6	40.92±1.76	57.29±2.73	315.8±12.5	58.49±2.01
[M-H]⁻	18:3	431.2205	431.2204	0.23	277.7±11.5	79.99±4.55	140.1±9.6	335.9±0.3	66.52±2.02
					PA				
[M-H]⁻	14:0/18:1	645.4506	645.4501	0.77	149.7±11.3	nd	nd	nd	nd
[M-H]⁻	15:0/18:1	659.4661	659.4657	0.61	140.0±11.3	7.27±0.24	46.93±3.49	nd	nd
[M-H]⁻	16:0/16:1	645.4501	645.4501	0.00	146.6±9.9	118.1±9.1	182.6±3.9	nd	nd
[M-H]⁻	16:0/18:1	673.4817	673.4814	0.45	168.3±3.9	132.2±6.3	198.1±5.1	nd	234.9±27.4
[M-H]⁻	16:0/18:3	669.4509	669.4501	1.20	7.79±0.53	111.4±10.6	13.18±0.62	nd	nd

[M-H]-	16:0/20:5	693.4504	693.4501	0.43	157.7±12.6	128.7±9.4	232.2±14.2	57.37±1.26	192.3±12.2
[M-H]-	16:0/22:6	719.4657	719.4657	0.00	4250±142	nd	nd	164.6±4.6	nd
[M-H]-	16:1/16:1	643.4348	643.4344	0.62	nd	nd	148.1±11.5	nd	nd
[M-H]-	16:1/18:1	671.4663	671.4657	0.89	148.0±8.6	124.9±7.9	165.1±4.6	nd	8.31±0.90
[M-H]-	16:1/20:5	691.4348	691.4344	0.58	144.4±6.4	20.38±0.62	164.6±12.2	nd	15.93±1.25
[M-H]-	16:1/22:6	717.4506	717.4501	0.70	147.4±10.2	nd	155.4±2.7	4.58±0.47	12.99±0.87
[M-H]-	17:0/18:1	687.4981	687.4970	1.60	133.4±10.4	6.65±0.40	16.83±1.10	nd	nd
[M-H]-	17:0/20:4	709.4822	709.4814	1.13	nd	105.5±8.2	nd	nd	nd
[M-H]-	17:0/20:5	707.4666	707.4657	1.27	153.2±9.0	12.53±0.75	142.6±12.5	4.69±0.38	178.0±17.2
[M-H]-	17:0/22:6	733.4818	733.4814	0.55	161.3±15.5	105.0±9.2	nd	nd	nd
[M-H]-	17:1/18:1	685.4813	685.4814	-0.15	133.7±10.4	109.5±8.5	141.1±9.2	nd	nd
[M-H]-	17:1/20:5	705.4510	705.4501	1.28	48.19±2.54	nd	142.4±6.2	nd	nd
[M-H]-	17:1/22:6	731.4666	731.4657	1.23	54.27±3.72	13.16±1.03	137.7±9.2	nd	nd
[M-H]-	18:0/20:4	723.4980	723.4970	1.38	nd	58.21±4.34	nd	nd	173.8±14.6
[M-H]-	18:0/20:5	721.4821	721.4814	0.97	1670±34	3.79±0.16	nd	168.0±3.7	170.0±8.6
[M-H]-	18:1/18:1	699.4976	699.4970	0.86	144.4±9.2	117.5±9.7	149.5±12.3	11.47±0.80	8.75±0.29
[M-H]-	18:1/18:2	697.4824	697.4814	1.43	11.64±0.52	112.6±8.7	22.53±1.61	nd	nd
[M-H]-	18:1/20:1	727.5293	727.5283	1.37	126.5±8.6	103.5±9.2	10.88±1.13	nd	nd
[M-H]-	18:1/20:5	719.4662	719.4657	0.69	290.7±28.6	169.5±12.9	257.7±16.8	nd	220.6±0.0
[M-H]-	18:1/22:6	745.4819	745.4814	0.67	230.8±20.2	179.0±4.5	193.2±7.9	nd	179.3±17.4
[M-H]-	18:2/20:5	717.4507	717.4501	0.84	nd	116.6±11.3	nd	nd	24.55±1.17
[M-H]-	18:2/22:6	743.4667	743.4657	1.35	125.9±10.1	124.6±4.3	nd	nd	3.61±0.33
[M-H]-	18:3/20:5	715.4353	715.4344	1.26	nd	108.1±7.5	nd	nd	nd
[M-H]-	18:3/22:6	741.4509	741.4501	1.08	84.00±6.97	113.4±3.9	nd	nd	4.58±0.40
[M-H]-	19:1/20:5	733.4822	733.4814	1.09	135.0±8.8	nd	141.1±13.8	23.08±1.64	17.73±1.08
[M-H]-	19:1/22:6	759.4978	759.4970	1.05	120.9±9.5	nd	126.5±9.8	31.07±1.37	nd
[M-H]-	20:1/22:6	773.5134	773.5127	0.90	121.5±8.8	nd	52.36±3.96	nd	nd
[M-H]-	20:3/20:5	743.4667	743.4657	1.35	nd	nd	139.1±6.1	nd	nd
[M-H]-	20:5/20:5	739.4349	739.4344	0.68	131.9±10.7	108.8±7.8	168.4±9.6	10.82±1.06	174.7±11.5
[M-H]-	20:5/21:5	753.4508	753.4501	0.93	nd	nd	124.7±9.6	nd	108.2±2.0
[M-H]-	20:5/22:6	765.4503	765.4501	0.26	131.0±8.8	125.2±9.6	159.9±13.1	51.02±2.82	167.1±13.9
[M-H]-	22:6/22:6	791.4665	791.4657	1.01	nd	105.2±9.5	121.1±9.3	nd	nd

LPI

[M-H]⁻	20:5	617.2732	617.2732	0.00	2.81±0.16	28.82±1.14	1.12±0.12	30.66±1.49	3.06±0.20
					PI				
[M-H]⁻	15:0/20:5	841.4877	841.4873	0.48	27.79±2.22	nd	28.64±2.35	nd	nd
[M-H]⁻	16:0/18:1	835.5349	835.5342	0.84	nd	21.38±0.87	nd	21.52±1.51	nd
[M-H]⁻	16:0/20:5	855.5034	855.5029	0.58	123.1±2.5	64.13±4.96	73.98±5.61	31.95±2.81	91.64±0.70
[M-H]⁻	16:1/20:5	853.4872	853.4873	-0.12	28.72±2.59	22.55±1.85	29.51±2.45	21.17±1.56	28.03±1.36
[M-H]⁻	17:0/20:5	869.5190	869.5186	0.46	41.43±3.58	32.30±1.69	45.74±4.03	24.84±2.34	48.25±0.14
[M-H]⁻	17:1/20:5	867.5034	867.5029	0.58	28.00±2.47	22.24±1.73	28.78±2.17	7.67±0.18	26.63±0.59
[M-H]⁻	18:0/18:1	863.5665	863.5655	1.16	nd	nd	1.1±0.080	20.73±1.41	1.19±0.14
[M-H]⁻	18:0/20:5	883.5341	883.5342	-0.11	nd	178.2±16.1	231.0±24.0	62.03±7.77	278.3±3.0
[M-H]⁻	18:0/22:6	909.5510	909.5499	1.21	nd	nd	nd	20.03±1.38	nd
[M-H]⁻	18:1/20:5	881.5190	881.5186	0.45	159.1±15.6	53.09±4.33	86.57±6.84	25.17±1.69	98.59±0.86
[M-H]⁻	18:2/20:5	879.5033	879.5029	0.45	27.52±1.74	24.99±2.18	28.83±2.80	20.51±1.40	27.63±0.46
[M-H]⁻	18:3/20:5	877.4881	877.4873	0.91	3.03±0.14	21.88±2.15	nd	nd	nd
[M-H]⁻	19:0/20:5	897.5502	897.5499	0.33	44.39±4.00	22.73±1.15	nd	20.17±1.35	nd
[M-H]⁻	19:1/20:5	895.5341	895.5342	-0.11	28.54±1.73	22.08±1.70	26.16±1.12	20.17±1.41	27.16±0.54
[M-H]⁻	20:1/20:5	909.5509	909.5499	1.10	nd	nd	nd	nd	26.68±0.68
[M-H]⁻	20:2/20:5	907.5345	907.5342	0.33	28.24±2.20	62.03±4.51	29.55±0.15	19.89±1.33	28.04±0.43
[M-H]⁻	20:4/20:5	903.5040	903.5029	1.22	nd	21.43±1.66	12.28±1.34	nd	nd
[M-H]⁻	20:5/20:5	901.4875	901.4873	0.22	26.81±2.40	21.61±1.75	27.96±2.71	20.29±1.47	26.52±0.68
[M-H]⁻	20:5/22:5	929.5186	929.5186	0.00	1.78±0.16	13.69±1.24	25.96±2.12	nd	24.68±0.49
[M-H]⁻	20:5/22:6	927.5034	927.5029	0.54	25.37±1.98	21.36±1.65	26.16±2.24	19.24±1.33	24.56±0.46
[M-H]⁻	20:4/18:2;O	897.5137	897.5135	0.22	25.96±2.02	70.16±5.86	nd	21.61±2.03	nd

nd: not detected

Table S5. Molecular species of lipids extracted from edible viscera of five kinds of crabs

Type	Species	m/z	m/z ref	ppm	Concentrations (nmol/g)				
					P. camtschaticus	*E. sinensis*	*C. magister*	*P. trituberculatus*	*C. pagurus*
					LPC				
$[M+H]^+$	16:0	496.3400	496.3398	0.40	67.19±3.01	8.92±0.45	290.9±7.9	436.4±4.2	175.6±4.1
$[M+H]^+$	16:1	494.3240	494.3241	-0.20	23.35±0.71	6.09±0.2	314.1±11.0	22.33±1.73	81.24±2.50
$[M+H]^+$	18:1	522.3554	522.3554	0.00	269.3±9.7	19.41±1.88	1040±41	1217±42	276.1±2.9
$[M+H]^+$	20:1	550.3868	550.3867	0.18	18.99±1.14	nd	38.07±3.27	29.45±0.69	16.68±0.98
$[M+H]^+$	20:4	544.3400	544.3398	0.37	nd	nd	nd	202.0±8.2	nd
$[M+H]^+$	20:5	542.3243	542.3241	0.37	67.51±5.45	4.16±0.18	684.7±31.8	305.9±23.4	321.4±16.2
$[M+H]^+$	22:6	568.3392	568.3398	-1.06	32.01±3.65	6.73±0.38	459.2±27.7	393.0±28.5	210.4±14.5
$[M+H]^+$	O-15:0	468.3452	468.3449	0.64	5.14±0.22	nd	8.26±0.31	23.48±0.74	12.56±0.95
$[M+H]^+$	O-16:1	480.3449	480.3449	0.00	26.70±2.09	1.80±0.09	nd	43.14±1.06	23.18±0.65
$[M+H]^+$	O-18:1	508.3765	508.3762	0.59	6.54±0.40	nd	16.65±0.71	131.7±3.4	20.33±2.85
					PC				
$[M+H]^+$	13:0/20:5	738.5074	738.5068	0.81	383.7±11.3	2.81±0.19	230.0±17.0	115.5±11.6	121.5±6.5
$[M+H]^+$	15:0/18:1	746.5692	746.5694	-0.27	136.6±2.5	30.10±1.03	740.6±14.8	294.7±10.5	459.2±37.8
$[M+H]^+$	15:0/20:1	774.6006	774.6007	-0.13	129.2±10.1	3.47±0.48	515.1±20.4	31.98±4.26	173.1±14.1
$[M+H]^+$	15:1/18:1	744.5541	744.5538	0.40	30.67±3.45	26.18±2.07	279.5±23.4	393.7±16.0	150.8±10.9
$[M+H]^+$	15:1/20:5	764.5228	764.5225	0.39	549.9±13.2	11.15±1.71	966.9±51.2	314.1±17.8	208.9±4.7
$[M+H]^+$	16:0/20:5	780.5537	780.5538	-0.13	1368±56	127.0±5.7	2691±210	118.6±15.3	1874±53
$[M+H]^+$	16:1/16:1	730.5378	730.5381	-0.41	nd	55.55±3.47	nd	115.4±5.4	2.51±0.42
$[M+H]^+$	16:1/18:1	758.5687	758.5694	-0.92	nd	534.1±5.7	6490±260	nd	nd
$[M+H]^+$	16:1/20:5	778.5375	778.5381	-0.77	nd	48.16±2.50	1272±79	148.4±9.7	123.9±7.4
$[M+H]^+$	17:1/18:1	772.5850	772.5851	-0.13	115.9±5.4	38.23±1.98	449.1±24.6	62.78±2.91	119.0±8.7
$[M+H]^+$	17:1/20:5	792.5549	792.5538	1.39	485.8±24.9	86.52±6.53	2191±187	565.0±10.8	104.9±8.4
$[M+H]^+$	18:0/20:2	814.6321	814.6320	0.12	262.7±3.4	nd	nd	14.29±1.70	nd
$[M+H]^+$	18:0/22:2	842.6617	842.6633	-1.90	13.16±0.69	nd	61.62±2.44	nd	nd
$[M+H]^+$	18:1/18:1	786.6014	786.6007	0.89	1086±65	258.3±19.1	5279±514	13.02±1.68	470.6±12.1
$[M+H]^+$	19:0/18:2	800.6158	800.6164	-0.75	17.89±2.11	13.01±1.61	151.6±10.4	3.81±0.16	8.53±0.41
$[M+H]^+$	20:5/20:5	826.5370	826.5381	-1.33	56.01±5.13	nd	nd	29.99±1.99	115.6±1.9
$[M+H]^+$	20:5/22:6	852.5522	852.5538	-1.88	55.81±3.10	18.66±1.51	144.3±2.4	28.74±2.05	114.6±0.8

$[M+H]^+$	22:2/18:5	832.5854	832.5851	0.36	1142±63	182.1±18.4	3047±80	nd	690.0±57.1
$[M+H]^+$	22:6/22:6	878.5698	878.5694	0.46	18.39±1.02	15.56±1.25	51.20±5.31	nd	40.60±2.76
					LPE				
$[M+H]^+$	20:5	500.2779	500.2772	1.40	100.6±5.2	15.79±0.99	448.2±14.1	86.53±2.76	157.5±13.2
$[M+H]^+$	22:6	526.2932	526.2928	0.76	44.73±3.81	14.77±0.98	286.4±26.1	85.67±4.38	115.8±9.4
$[M+H]^+$	O-18:1	466.3291	466.3292	-0.21	144.0±11.2	nd	176.6±10.2	193.3±10.8	105.8±7.4
$[M+H]^+$	16:0/16:1	690.5061	690.5068	-1.01	69.36±3.48	32.45±2.15	198.4±9.3	10.49±0.71	60.90±3.06
$[M+H]^+$	16:0/18:2	716.5218	716.5225	-0.98	76.25±7.00	116.3±9.0	215.3±23.6	19.22±1.80	45.09±3.56
$[M+H]^+$	18:0/18:5	738.5074	738.5068	0.81	316.2±31.9	135.6±12.4	1176±99	nd	482.9±10.5
$[M+H]^+$	18:0/20:5	766.5381	766.5381	0.00	8.33±0.66	84.43±0.64	1394±20	64.68±3.93	367.8±19.1
$[M+H]^+$	18:1/20:5	764.5212	764.5225	-1.70	2080±50	461.5±25.0	808.1±57.0	54.86±4.53	917.7±16.9
$[M+H]^+$	20:1/22:6	818.5682	818.5694	-1.47	32.39±3.53	nd	90.57±5.70	nd	nd
$[M+H]^+$	20:2/20:2	796.5840	796.5851	-1.38	nd	20.68±1.80	267.4±18.5	7.14±0.31	nd
$[M+H]^+$	20:2/20:5	790.5378	790.5381	-0.38	579.4±21.2	183.9±8.7	256.9±14.3	14.84±1.34	320.5±17.8
$[M+H]^+$	P-16:0/20:5	722.5120	722.5119	0.14	1001±49	176.5±11.2	839.6±26.8	164.3±5.6	63.38±4.75
$[M+H]^+$	P-16:0/22:6	748.5275	748.5276	-0.13	nd	154.9±6.1	3069±258	572.5±0.5	1389±91
$[M+H]^+$	P-17:0/22:6	762.5427	762.5432	-0.66	155.1±3.9	128.2±9.7	1268±7	244.2±8.6	118.3±3.3
$[M+H]^+$	P-18:0/20:5	750.5439	750.5432	0.93	3329±121	368.8±17.3	7243±215	1221±14	3156±131
$[M+H]^+$	P-18:0/22:6	776.5593	776.5589	0.52	1621±54	447.9±33.6	10250±677	1227±39	1818±96
$[M+H]^+$	P-18:1/22:6	774.5438	774.5432	0.77	218.0±19.0	66.47±3.01	2263±29	337.7±28.5	616.0±2.5
$[M+H]^+$	P-19:0/22:6	790.5745	790.5745	0.00	22.81±2.36	nd	152.2±13.6	42.67±5.31	54.42±3.46
$[M+H]^+$	P-20:1/22:6	802.5746	802.5745	0.12	145.7±9.4	29.54±2.62	1421±11	16.47±1.39	111.1±5.1
					TAG				
$[M+NH_4]^+$	12:0/16:0/16:1	766.6911	766.6919	-1.04	33.37±1.57	483.1±12.5	92.02±7.40	119.4±8.4	24.18±0.67
$[M+NH_4]^+$	12:0/16:1/18:1	792.7076	792.7076	0.00	73.00±1.17	2356±203	505.7±23.6	49.37±2.71	54.96±1.19
$[M+NH_4]^+$	14:0/14:0/16:0	768.7051	768.7076	-3.25	16.95±1.65	267.2±17.7	52.74±0.71	250.9±2.4	23.36±1.68
$[M+NH_4]^+$	14:0/15:0/16:0	782.7218	782.7232	-1.79	nd	206.4±7.6	42.73±1.27	172.6±4.3	8.98±0.92
$[M+NH_4]^+$	14:0/16:0/16:0	796.7369	796.7389	-2.51	nd	nd	nd	673.8±27.6	89.90±7.78
$[M+NH_4]^+$	14:0/16:0/16:1	794.7230	794.7232	-0.25	169.9±7.5	2139±84	639.8±47.4	450.6±5.3	118.1±9.8
$[M+NH_4]^+$	14:0/16:0/18:1	822.7551	822.7545	0.73	161.4±12.8	52.17±2.29	471.2±27.6	2193±70	136.5±4.8
$[M+NH_4]^+$	14:0/16:1/18:1	820.7391	820.7389	0.24	1028±90	2790±198	nd	268.5±18.9	74.53±3.07
$[M+NH_4]^+$	14:0/18:0/18:1	850.7853	850.7858	-0.59	204.9±11.8	6044±228	436.4±13.3	1085±80	30.76±2.32
$[M+NH_4]^+$	15:0/16:0/16:1	808.7365	808.7389	-2.97	nd	nd	nd	320.2±24.3	nd

$[M+NH_4]^+$	15:0/17:1/21:5	882.7541	882.7545	-0.45	nd	nd	nd	nd	92.52±1.23
$[M+NH_4]^+$	16:0/14:1/18:2	818.7227	818.7232	-0.61	nd	5765±85	nd	nd	nd
$[M+NH_4]^+$	16:0/16:0/20:5	870.7534	870.7545	-1.26	128.2±10.4	14410±879	1755±174	135.2±8.1	500.1±10.1
$[M+NH_4]^+$	16:0/16:0/22:6	896.7678	896.7702	-2.68	nd	nd	16.05±0.99	35.81±0.70	387.3±14.7
$[M+NH_4]^+$	16:0/16:1/18:1	848.7704	848.7702	0.24	240.9±11.7	294.2±10.5	841.0±76.5	465.3±0.6	191.0±7.5
$[M+NH_4]^+$	16:0/16:1/18:4	842.7234	842.7232	0.24	251.9±17.7	nd	186.0±18.4	28.61±1.61	67.57±6.28
$[M+NH_4]^+$	16:0/16:1/20:1	876.8014	876.8015	-0.11	1343±69	10320±359	249.6±10.1	567.9±31.4	437.4±10.9
$[M+NH_4]^+$	16:0/17:0/16:1	836.7698	836.7702	-0.48	14.26±0.97	2325±200	49.43±1.54	122.1±9.8	nd
$[M+NH_4]^+$	16:0/17:0/18:1	864.8003	864.8015	-1.39	42.04±2.20	1168±140	46.50±2.42	129.7±16.1	40.86±1.67
$[M+NH_4]^+$	16:0/18:1/18:3	872.7703	872.7702	0.11	nd	293.2±25.5	nd	nd	nd
$[M+NH_4]^+$	16:0/18:1/19:1	890.8163	890.8171	-0.90	84.77±8.35	1333±121	133.7±7.4	343.1±16.1	105.5±5.8
$[M+NH_4]^+$	16:0/18:1/20:1	904.8327	904.8328	-0.11	1689±3	3871±21	714.5±21.7	372.8±15.2	111.2±6.2
$[M+NH_4]^+$	16:0/18:1/22:1	932.8632	932.8641	-0.96	874.6±64.5	824.0±1.9	359.9±14.4	61.64±3.40	257.8±17.5
$[M+NH_4]^+$	16:0/20:1/18:2	902.8157	902.8171	-1.55	3984±97	265.5±11.9	528.5±4.8	0.60±0.05	nd
$[M+NH_4]^+$	16:0/20:1/20:2	930.8475	930.8484	-0.97	372.8±14.4	431.6±39.2	386.1±14.6	nd	138.2±12.1
$[M+NH_4]^+$	16:0/20:1/22:1	960.8935	960.8954	-1.98	356.5±14.5	1128±57	154.4±14.2	55.90±1.71	172.1±11.9
$[M+NH_4]^+$	16:0/20:1/24:1	988.9267	988.9267	0.00	130.1±9.8	209.9±18.4	118.5±8.8	35.54±2.53	24.96±1.45
$[M+NH_4]^+$	16:0/24:0/18:1	962.9087	962.9110	-2.39	46.86±4.32	616.1±18.2	179.2±7.1	48.57±3.25	43.95±2.03
$[M+NH_4]^+$	16:1/16:1/18:1	846.7544	846.7545	-0.12	650.5±62.1	134.6±7.8	3087±92	nd	95.12±12.36
$[M+NH_4]^+$	16:1/18:1/18:4	868.7396	868.7389	0.81	466.1±20.2	2792±106	473.0±23.4	22.83±1.02	96.63±12.31
$[M+NH_4]^+$	16:1/18:1/22:1	930.8467	930.8484	-1.83	608.0±18.5	1524±111	233.5±18.9	nd	204.0±11.5
$[M+NH_4]^+$	16:1/20:1/22:1	958.8796	958.8797	-0.10	986.4±122.6	667.0±47.4	234.4±19.4	nd	189.8±10.7
$[M+NH_4]^+$	17:0/18:1/22:5	938.8156	938.8171	-1.60	nd	94.85±9.92	24.44±1.77	26.66±1.66	139.5±3.1
$[M+NH_4]^+$	17:1/18:1/20:1	916.8310	916.8328	-1.96	271.1±30.7	nd	294.1±12.8	6.26±0.31	78.02±6.73
$[M+NH_4]^+$	18:1/18:1/22:6	948.7990	948.8015	-2.63	953.6±109.9	1167±106	259.3±6.9	48.06±1.59	48.63±2.05
$[M+NH_4]^+$	18:1/18:3/14:1;1O	858.7187	858.7181	0.70	nd	67.53±1.37	nd	12.03±0.85	nd
$[M+NH_4]^+$	18:1/20:1/22:1	986.9113	986.9110	0.30	380.4±26.1	290.6±17.8	112.0±14.3	12.41±1.11	44.30±2.06
$[M+NH_4]^+$	18:1/20:1/24:1	1014.9410	1014.9423	-1.28	151.2±3.7	119.4±4.3	55.86±1.82	nd	12.20±0.82
$[M+NH_4]^+$	18:1/22:1/24:1	1042.9710	1042.9736	-2.49	90.02±8.97	nd	nd	nd	nd
					DAG				
$[M+NH_4]^+$	16:0/20:5	632.5244	632.5249	-0.79	619.4±69.9	nd	226.5±10.1	58.68±4.14	506.1±12.3
$[M+NH_4]^+$	16:0/22:6	658.5399	658.5405	-0.91	620.7±43.7	506.2±9.7	530.1±17.3	nd	1159±67
$[M+NH_4]^+$	18:1/18:3	634.5406	634.5405	0.16	72.99±5.31	469.7±27.2	nd	19.43±0.97	50.97±3.50

$[M+NH_4]^+$	O-15:0/14:1	528.4987	528.4986	0.19	119.9±8.3	173.5±20.3	29.73±3.10	789.8±34.5	28.09±1.62
$[M+NH_4]^+$	O-17:0/14:1	556.5299	556.5299	0.00	1381±60	2829±84	1066±32	248.2±18.2	441.1±2.1
					LPG				
$[M-H]^-$	15:0	469.2575	469.2572	0.64	nd	1.09±0.15	nd	61.06±0.85	11.48±1.10
$[M-H]^-$	16:1	481.2574	481.2572	0.42	nd	nd	nd	59.45±0.70	nd
$[M-H]^-$	18:1	509.2890	509.2885	0.98	nd	nd	nd	57.46±1.53	nd
$[M-H]^-$	20:2	535.3041	535.3041	0.00	nd	nd	nd	56.33±2.43	nd
$[M-H]^-$	20:5	529.2580	529.2572	1.51	nd	nd	nd	54.26±0.12	nd
$[M-H]^-$	22:6	555.2730	555.2728	0.36	nd	nd	nd	54.48±1.38	nd
$[M-H]^-$	O-15:4	447.2169	447.2153	3.58	85.84±4.29	3.95±0.06	1.68±0.11	65.32±1.65	nd
$[M-H]^-$	O-24:6	569.3235	569.3249	-2.46	nd	nd	nd	53.71±0.53	nd
					PG				
$[M-H]^-$	15:0/15:0	693.4713	693.4712	0.14	nd	nd	nd	nd	35.77±0.38
$[M-H]^-$	16:0/18:1	747.5182	747.5182	0.00	50.02±1.76	nd	27.86±0.99	14.17±0.92	60.67±3.47
$[M-H]^-$	16:0/20:2	773.5338	773.5338	0.00	48.07±1.54	nd	28.89±1.07	37.52±0.29	49.58±2.49
$[M-H]^-$	16:0/20:4	769.5034	769.5025	1.17	9.57±0.45	nd	11.51±1.22	nd	33.91±1.11
$[M-H]^-$	16:1/20:4	767.4872	767.4869	0.39	5.96±0.21	nd	27.44±0.38	nd	44.22±3.12
$[M-H]^-$	18:0/20:5	795.5191	795.5182	1.13	3.85±0.16	nd	27.22±0.69	nd	31.31±0.56
$[M-H]^-$	18:1/20:5	793.5032	793.5025	0.88	nd	nd	26.48±0.77	nd	13.42±0.56
					CL				
$[M-2H]^{2-}$	16:0/16:1/18:1/20:3	712.4847	712.4867	-2.81	5.45±0.45	nd	nd	nd	17.37±1.16
$[M-2H]^{2-}$	16:0/18:1/18:1/18:1	714.5085	714.5023	8.68	10.00±0.32	nd	66.75±1.76	nd	nd
$[M-2H]^{2-}$	16:1/16:1/18:1/18:1	699.4788	699.4788	0.00	4.58±0.30	nd	1.98±0.18	nd	2.86±0.11
$[M-2H]^{2-}$	16:1/18:1/18:1/18:1	713.4952	713.4945	0.98	1.24±0.12	nd	1.57±0.07	nd	nd
$[M-2H]^{2-}$	16:1/18:1/18:1/20:3	725.4929	725.4945	-2.21	6.99±0.12	nd	1.62±0.16	nd	nd
$[M-2H]^{2-}$	16:1/18:1/18:1/20:5	723.4788	723.4788	0.00	33.83±2.35	nd	67.44±5.02	1.84±0.11	32.37±2.36
$[M-2H]^{2-}$	16:1/18:1/20:3/20:3	737.4955	737.4945	1.36	73.94±3.96	nd	145.5±5.8	nd	nd
$[M-2H]^{2-}$	16:1/18:2/20:5/22:6	745.4623	745.4632	-1.21	nd	nd	nd	nd	33.58±0.28
$[M-2H]^{2-}$	16:1/20:1/20:5/20:5	747.4788	747.4788	0.00	11.4±0.9	nd	nd	nd	nd
$[M-2H]^{2-}$	16:1/20:3/20:3/20:3	749.4943	749.4945	-0.27	nd	nd	nd	nd	35.94±0.75
$[M-2H]^{2-}$	17:1/17:1/17:1/18:2	705.4871	705.4788	11.77	7.90±0.50	5.52±0.37	12.29±1.12	nd	11.48±0.78
$[M-2H]^{2-}$	18:0/18:1/18:1/18:3	726.5018	726.5023	-0.69	12.75±1.72	nd	16.88±2.06	nd	9.80±1.49
$[M-2H]^{2-}$	18:0/18:1/20:4/22:4	764.5252	764.5180	9.42	nd	nd	nd	nd	261.5±14.5

$[M-2H]^{2-}$	18:0/18:1/20:5/22:5	762.5086	762.5023	8.26	256.0±26.0	nd	18.62±1.44	nd	276.0±9.3
$[M-2H]^{2-}$	18:0/20:3/22:6/22:6	786.5027	786.5023	0.51	nd	nd	nd	nd	9.15±0.44
$[M-2H]^{2-}$	18:0/20:4/22:6/22:6	785.4921	785.4945	-3.06	nd	nd	nd	nd	9.52±0.49
$[M-2H]^{2-}$	18:0/24:3/20:5/22:5	802.5394	802.5336	7.23	nd	nd	3.51±0.08	nd	63.03±0.01
$[M-2H]^{2-}$	18:1/18:1/18:2/20:5	736.4912	736.4867	6.11	75.73±3.07	nd	351.2±13.5	nd	115.6±9.3
$[M-2H]^{2-}$	18:1/20:2/20:5/20:6	759.4791	759.4788	0.40	nd	nd	11.83±0.93	nd	53.01±1.53
$[M-2H]^{2-}$	18:1/20:2/20:6/22:6	772.4861	772.4867	-0.78	nd	nd	nd	nd	33.52±1.04
$[M-2H]^{2-}$	18:1/20:3/20:3/20:5	761.4956	761.4945	1.44	6.21±0.56	nd	nd	nd	33.84±0.75
$[M-2H]^{2-}$	18:1/20:4/20:5/22:6	771.4778	771.4788	-1.30	nd	nd	4.35±0.24	nd	17.72±0.95
$[M-2H]^{2-}$	18:1/20:5/22:6/22:6	783.4784	783.4788	-0.51	nd	nd	3.15±0.08	nd	31.68±0.56
$[M-2H]^{2-}$	24:0/16:1/16:1/18:1	742.5401	742.5336	8.75	9.49±0.66	nd	nd	nd	2.93±0.73
$[M-2H]^{2-}$	24:0/18:1/20:5/20:5	790.5402	790.5336	8.35	21.39±2.15	nd	66.66±3.41	nd	25.08±1.35
					SM				
$[M+HCOO]^{-}$	12:1;2O/18:0	691.5031	691.5032	-0.14	1032±47	1108±10	57.63±3.18	134.2±8.4	694.6±16.7
$[M+HCOO]^{-}$	12:1;2O/21:0	733.5507	733.5501	0.82	141.4±5.6	nd	569.9±25.3	773.4±3.2	nd
$[M+HCOO]^{-}$	12:1;2O/24:1	773.5820	773.5814	0.78	1172±79	nd	765.7±30.2	34.34±2.86	184.7±7.3
$[M+HCOO]^{-}$	14:1;2O/18:0	719.5349	719.5345	0.56	998.9±46.5	nd	1106±36	856.8±10.1	701.6±15.6
$[M+HCOO]^{-}$	14:1;2O/20:0	747.5669	747.5658	1.47	617.0±11.8	nd	643.6±28.7	850.9±25.1	667.9±14.6
$[M+HCOO]^{-}$	14:1;2O/21:0	761.5832	761.5814	2.36	nd	nd	nd	730.0±5.5	71.14±2.73
$[M+HCOO]^{-}$	14:1;2O/22:0	775.5981	775.5971	1.29	nd	nd	9.63±1.46	754.9±8.6	662.4±17.4
					PA				
$[M-H]^{-}$	16:0/16:1	645.4501	645.4501	0.00	18.41±1.31	nd	469.1±15.1	nd	nd
$[M-H]^{-}$	16:0/18:1	673.4819	673.4814	0.74	238.3±20.5	nd	472.6±17.5	nd	178.4±9.3
$[M-H]^{-}$	16:0/20:5	693.4506	693.4501	0.72	148.7±8.4	nd	434.6±16.1	nd	529.4±2.1
$[M-H]^{-}$	16:1/18:1	671.4662	671.4657	0.74	21.93±1.69	nd	450.6±10.6	nd	537.0±13.1
$[M-H]^{-}$	18:0/20:5	721.4819	721.4814	0.69	765.2±44.5	nd	509.1±13.1	nd	580.3±39.3
$[M-H]^{-}$	18:1/18:1	699.4974	699.4970	0.57	nd	nd	435.9±11.9	nd	111.3±9.0
$[M-H]^{-}$	18:1/20:1	727.5295	727.5283	1.65	17.54±1.14	nd	399.3±1.7	nd	nd
$[M-H]^{-}$	18:1/20:5	719.4662	719.4657	0.69	750.6±41.4	nd	439.1±17.9	nd	533.5±1.3
$[M-H]^{-}$	18:1/22:6	745.4818	745.4814	0.54	730.4±16.1	nd	406.5±7.9	15.96±1.54	417.0±18.2
$[M-H]^{-}$	18:2/18:2	695.4662	695.4657	0.72	598.3±28.6	nd	nd	49.54±3.27	nd
$[M-H]^{-}$	20:5/22:6	765.4503	765.4501	0.26	nd	nd	nd	nd	77.50±6.72
					LPI				

[M-H]$^-$	18:1	597.3051	597.3045	1.00	1.83±0.04	nd	nd	nd	100.4±0.1
					PI				
[M-H]$^-$	16:0/20:5	855.5036	855.5029	0.82	nd	nd	201.0±13.6	205.3±8.8	nd
[M-H]$^-$	16:1/18:1	833.5196	833.5186	1.20	nd	nd	62.13±1.06	nd	nd
[M-H]$^-$	16:1/20:5	853.4878	853.4873	0.59	111.6±3.8	nd	33.22±2.67	nd	77.7±3.1
[M-H]$^-$	17:0/20:2	875.5657	875.5655	0.23	nd	nd	58.64±1.51	nd	nd
[M-H]$^-$	17:0/20:5	869.5190	869.5186	0.46	111.4±1.3	6.57±0.39	66.98±2.29	82.03±0.19	103.6±8.6
[M-H]$^-$	17:1/18:1	847.5348	847.5342	0.71	nd	nd	59.02±0.12	nd	nd
[M-H]$^-$	17:1/20:5	867.5039	867.5029	1.15	nd	nd	60.03±0.91	nd	2.73±0.22
[M-H]$^-$	18:0/20:5	883.5354	883.5342	1.36	nd	34.47±2.42	nd	94.30±7.90	163.9±9.2
[M-H]$^-$	18:1/18:1	861.5511	861.5499	1.39	109.8±3.5	nd	nd	nd	nd
[M-H]$^-$	18:1/20:4;O	899.5298	899.5291	0.78	nd	nd	nd	nd	4.64±0.36
[M-H]$^-$	18:1/20:5	881.5190	881.5186	0.45	210.0±10.2	112.6±2.0	221.6±21.8	110.7±12.5	186.4±8.0
[M-H]$^-$	18:1/20:5;O	897.5137	897.5135	0.22	115.8±8.3	nd	nd	nd	1.75±0.07
[M-H]$^-$	18:1/22:6	907.5344	907.5342	0.22	109.2±5.5	108.2±0.4	73.48±1.88	nd	nd
[M-H]$^-$	18:2/20:5	879.5032	879.5029	0.34	107.3±2.8	111.2±0.2	60.88±1.94	nd	74.45±2.01
[M-H]$^-$	19:0/20:5	897.5504	897.5499	0.56	108.7±4.1	nd	nd	nd	15.31±0.90
[M-H]$^-$	19:1/20:5	895.5348	895.5342	0.67	108.4±3.1	1.18±0.15	62.30±1.54	15.28±1.10	70.32±1.71
[M-H]$^-$	20:1/20:5	909.5505	909.5499	0.66	127.4±14.1	nd	44.44±2.17	27.82±1.50	nd
[M-H]$^-$	20:2/20:5	907.5349	907.5342	0.77	nd	nd	nd	nd	76.02±3.10
[M-H]$^-$	20:4/18:2;O	897.5146	897.5135	1.23	nd	nd	nd	nd	69.80±1.77
[M-H]$^-$	20:4/20:5	903.5044	903.5029	1.66	nd	nd	nd	4.65±0.29	68.74±0.48
[M-H]$^-$	20:5/20:5	901.4877	901.4873	0.44	298.0±11.65	nd	57.51±1.04	3.28±0.13	2.48±0.11
[M-H]$^-$	22:2/20:5	935.5665	935.5655	1.07	nd	nd	1.14±0.14	nd	65.42±1.79
[M-H]$^-$	O-16:0/20:5	841.4875	841.4873	0.24	148.1±12.4	114.6±1.8	134.6±11.8	85.52±1.61	216.2±4.9
[M-H]$^-$	O-18:0/20:5	869.5549	869.5549	0.00	nd	nd	62.26±2.04	nd	nd
[M-H]$^-$	O-18:1/20:5	867.5394	867.5393	0.12	216.0±4.4	nd	nd	nd	nd

nd: not detected

Table S6. Concentrations of different lipid classes in five kinds of crab muscles (ng/kg)

**Species**	***P. camtschaticus***	***E. sinensis***	***C. magister***	***P. trituberculatus***	***C. pagurus***
LPC	225.7±30.6^{bc}	316.2±9.1^{b}	222.3±2.2^{bc}	1721±89^{a}	132.3±0.9^{c}
PC	7522±160^{a}	2262±50^{d}	4610±114^{b}	1153±51^{e}	3438±77^{c}
LPE	123.8±9.2^{d}	269.5±15.2^{b}	168.0±5.2^{c}	1141±22^{a}	87.24±1.06^{e}
PE	11218±460^{b}	20459±309^{a}	8997±143^{c}	4657±66^{e}	7704±124^{d}
TAG	6284±257^{a}	5619±389^{b}	1626±48^{c}	1719±168^{c}	1884±85^{c}
DAG	1500±66^{a}	450.2±14.1^{b}	288.8±3.8^{c}	86.05±10.95^{d}	133.2±2.8^{d}
SM	3416±158^{a}	1613±45^{c}	1598±36^{c}	2350±42^{b}	869.9±22.5^{d}
CL	124.3±5.2^{d}	191.8±26.4^{cd}	817.9±38.7^{a}	169.0±7.8^{c}	528.3±21.0^{b}
LPG	nd	nd	nd	61.98±1.84^{a}	nd
PG	75.89±4.37^{c}	85.96±2.74^{c}	166.7±9.7^{a}	142.2±3.0^{b}	163.7±10.5^{a}
LPA	176.5±9.9^{b}	51.24±1.77^{d}	83.84±4.28^{c}	274.1±4.1^{a}	52.62±1.38^{d}
PA	6783±228^{a}	1815±95.79^{c}	2531±111^{b}	381.6±8.17^{e}	1365±55^{d}
LPI	1.73±0.08^{cd}	17.79±0.58^{b}	0.69±0.06^{d}	18.92±0.75^{a}	1.89±0.10^{c}
PI	544.3±29.7^{b}	615.5±34.5^{a}	619.0±35.6^{a}	332.5±18.1^{c}	669.1±1.0^{a}

nd means not detected; Data are expressed as the mean ± SD. Different letters in the same row indicate significant difference ($p < 0.05$) among different samples.

Table S7. The concentrations of different lipid classes of five kinds of edible viscera (ng/kg)

**Species**	***P. camtschaticus***	***E. sinensis***	***C. magister***	***P. trituberculatus***	***C. pagurus***
LPC	269.4 ± 7.8^{c}	24.53 ± 1.10^{d}	1508 ± 48^{a}	1477 ± 48^{a}	601.9 ± 18.1^{b}
PC	4624 ± 129^{b}	1130 ± 38^{e}	19196 ± 313^{a}	1726 ± 67^{d}	3755 ± 80^{c}
LPE	141.0 ± 7.9^{c}	15.67 ± 0.83^{d}	457.3 ± 10.5^{a}	178.5 ± 5.1^{b}	189.1 ± 10.9^{b}
PE	7319 ± 105^{b}	1826 ± 66^{d}	23615 ± 394^{a}	3040 ± 6^{c}	7234 ± 38^{b}
TAG	14260 ± 68^{b}	55795 ± 1378^{a}	11075 ± 182^{c}	6796 ± 129^{d}	3591 ± 61^{e}
DAG	1678 ± 30^{b}	2297 ± 45^{a}	1101 ± 16^{d}	604.9 ± 8.3^{e}	1376 ± 37^{c}
LPG	38.39 ± 1.57^{b}	2.28 ± 0.07^{d}	0.75 ± 0.04^{d}	235.4 ± 1.7^{a}	5.38 ± 0.42^{c}
PG	89.58 ± 2.09^{c}	nd	115.7 ± 1.3^{b}	39.62 ± 0.61^{d}	204.1 ± 6.0^{a}
CL	402.2 ± 8.9^{c}	3.89 ± 0.21^{d}	571.5 ± 17.4^{b}	1.33 ± 0.07^{d}	818.1 ± 13.9^{a}
SM	2904 ± 46^{b}	766.1 ± 5.9^{e}	2334 ± 71^{c}	3080 ± 22^{a}	2223 ± 43^{b}
PA	2355 ± 24^{b}	nd	2806 ± 42^{a}	46.35 ± 2.80^{d}	2098 ± 15^{c}
LPI	1.09 ± 0.02^{b}	nd	nd	nd	59.94 ± 0.03^{a}
PI	1660 ± 32^{a}	428.9 ± 4.9^{e}	1095 ± 26^{c}	545.9 ± 15.1^{b}	1186 ± 25^{d}

nd means not detected; Data are expressed as the mean ± SD. Different letters in the same row indicate significant difference ($p < 0.05$) among different samples.

Table S8. The significantly different lipids in muscle and edible viscera of five crab species

Muscle			Edible Viscera		
Compounds	VIP	*p* value	Compounds	VIP	*p* value
PE 16:0/22:6	3.35	＜0.05	TAG 16:0/20:1/18:2	3.15	＜0.01
PE P-18:0/20:5	2.86	＜0.05	SM 14:1;2O/22:0	1.85	＜0.01
PA 16:0/22:6	2.68	＜0.01	PE 18:1/20:5	1.78	＜0.05
PC 16:0/16:1	2.39	＜0.05	TAG 16:0/18:1/20:1	1.63	＜0.01
TAG 16:0/16:1/18:1	2.23	＜0.05	TAG 16:0/16:0/22:6	1.56	＜0.01
PE P-20:1/20:5	2.15	＜0.05	PA 16:1/18:1	1.49	＜0.05
PE P-19:0/20:4	1.86	＜0.01	SM 12:1;2O/18:0	1.44	＜0.05
SM 14:1;2O/18:0	1.86	＜0.05	TAG 14:0/16:1/18:1	1.42	＜0.05
PA 18:0/20:5	1.65	＜0.05	SM 12:1;2O/24:1	1.41	＜0.05
PC 22:2/18:5	1.60	＜0.05	PE P-16:0/20:5	1.35	＜0.01
SM 12:1;2O/20:0	1.59	＜0.01	CL 18:0/18:1/20:4/22:4	1.31	＜0.01
CL 18:1/18:2/22:6/22:6	1.49	＜0.01	PA 18:2/18:2	1.25	＜0.01
DAG 18:1/20:5	1.44	＜0.01	DAG O-17:0/14:1	1.25	＜0.01
PE 18:1/22:6	1.38	＜0.05	TAG 16:1/20:1/22:1	1.22	＜0.01
TAG 18:2/18:2/18:2	1.37	＜0.01	TAG 18:1/18:1/22:6	1.21	＜0.01
PA 18:0/20:4	1.36	＜0.05	TAG 16:0/18:1/22:1	1.02	＜0.01
TAG 18:1/18:1/20:5	1.33	＜0.01	LPC 16:0	1.01	＜0.05
TAG 18:1/18:1/20:1	1.30	＜0.01			
PC 15:1/20:5	1.29	＜0.01			
PC 18:1/18:1	1.29	＜0.01			
SM 14:1;2O/22:0	1.13	＜0.01			
DAG 16:0/20:5	1.10	＜0.01			
PC 17:1/18:1	1.04	＜0.05			
PI 18:0/20:5	1.03	＜0.05			
CL 18:1/20:5/22:6/22:6	1.02	＜0.01			
PE P-20:1/22:6	1.01	＜0.05			
